# Learning from small medical data—robust semi-supervised cancer prognosis classifier with Bayesian variational autoencoder

**DOI:** 10.1093/bioadv/vbac100

**Published:** 2023-01-09

**Authors:** Te-Cheng Hsu, Che Lin

**Affiliations:** Institute of Communications Engineering, National Tsing Hua University, Hsinchu 30013, Taiwan; Graduate Institute of Communication Engineering, National Taiwan University, Taipei 10617, Taiwan; Department of Electrical Engineering, National Taiwan University, Taipei 10617, Taiwan

## Abstract

**Motivation:**

Cancer is one of the world’s leading mortality causes, and its prognosis is hard to predict due to complicated biological interactions among heterogeneous data types. Numerous challenges, such as censorship, high dimensionality and small sample size, prevent researchers from using deep learning models for precise prediction.

**Results:**

We propose a robust Semi-supervised Cancer prognosis classifier with bAyesian variational autoeNcoder (*SCAN*) as a structured machine-learning framework for cancer prognosis prediction. *SCAN* incorporates semi-supervised learning for predicting 5-year disease-specific survival and overall survival in breast and non-small cell lung cancer (NSCLC) patients, respectively. *SCAN* achieved significantly better AUROC scores than all existing benchmarks (81.73% for breast cancer; 80.46% for NSCLC), including our previously proposed bimodal neural network classifiers (77.71% for breast cancer; 78.67% for NSCLC). Independent validation results showed that *SCAN* still achieved better AUROC scores (74.74% for breast; 72.80% for NSCLC) than the bimodal neural network classifiers (64.13% for breast; 67.07% for NSCLC). *SCAN* is general and can potentially be trained on more patient data. This paves the foundation for personalized medicine for early cancer risk screening.

**Availability and implementation:**

The source codes reproducing the main results are available on GitHub: https://gitfront.io/r/user-4316673/36e8714573f3fbfa0b24690af5d1a9d5ca159cf4/scan/.

**Supplementary information:**

Supplementary data are available at *Bioinformatics Advances* online.

## 1 Introduction

Cancer is one of the top leading causes of mortality. Lung and breast cancers are the most commonly diagnosed ones (Ferlay *et al.*, 2015). Lung cancer is the most prevalent cancer in men, where non-small cell lung cancer (NSCLC) accounts for approcimately 85% of lung cancer diagnoses (Ferlay *et al.*, 2015). The 5-year survival rate of lung cancer is less than 20% (Hirsch *et al.*, 2017), and the benefit of chemotherapy is significant in the early stages (Pignon *et al.*, 2008). Adjuvant treatments, such as post-operative cisplatin-based chemotherapy significantly improve NSCLC patient survival (Pignon *et al.*, 2008). Therefore, strong models for prognostic stratification are essential to help doctors identify potential high-risk patients for consequent therapeutic strategies. On the other hand, breast cancer is the most diagnosed cancer among females. It accounts for the second-leading cause of death in the USA for females (Siegel *et al.*, 2019). Due to the heterogeneity of breast cancer, variations in transcriptional programs and histology and molecular profiles are essential factors related to prognosis (Perou *et al.*, 2000). Reliable breast cancer prognosis prediction models are thus of crucial importance that can potentially reduce the suffering of the patients.

Several biomarkers were identified for both NSCLC and breast cancer as strong predictors for cancer prognosis prediction during the past decades. For instance, EPCAM, HIF1A, PKM, PTK7, ALCAM, CAMD1 and SLC2A1 were identified as well-known biomarkers (Baeuerle and Gires, 2007; Barron *et al.*, 2016; Chen *et al.*, 2014; Lau *et al.*, 2007; Münsterberg *et al.*, 2020; Papadaki *et al.*, 2014; Zeng *et al.*, 2015) for lung cancers. For breast cancer, ER, PR, HER2, Ki67 and uPA/PAI-1 are some of the well-known biomarkers (Carey *et al.*, 2007; Dent *et al.*, 2007; Dunnwald *et al.*, 2007; Lehmann *et al.*, 2011). In addition to these well-known biomarkers, we systematically analyzed potential gene candidates in our previous research and selected a small set of prognostic biomarkers. Along with the patient’s clinical information, we applied deep learning models to capture the complex multi-gene cross-talk interactions for predicting 5-year cancer patient survival (Cheng *et al.*, 2021; Lai *et al.*, 2020). In particular, we built gene interaction networks (Lai *et al.*, 2020) for candidate gene biomarker selection. We accurately predicted the 5-year overall survival (OS) and disease-specific survival (DSS) for NSCLC and breast cancer patients.

Several problems often arise when applying deep learning in biological applications, and many remain active research topics. These topics include approaches dealing with data scarcity (AbuKhousa *et al.*, 2012; Hsu and Lin, 2020, 2021), missing values (Che *et al.*, 2018; Fortuin *et al.*, 2020; Futoma *et al.*, 2017), combining heterogeneous data types (Gao *et al.*, 2020; Hügle *et al.*, 2021; Ravì *et al.*, 2017; Sun *et al.*, 2019) and model robustness (Ben Brahim and Limam, 2018; Cheng *et al.*, 2021; Dusenberry *et al.*, 2020; Shickel *et al.*, 2018). Although genetic data contain enormous amounts of information, they are, in practice, hard to be utilized efficiently by deep learning models before appropriate feature selection/engineering to prevent over-fitting. This is also known as the curse of dimensionality (Indyk and Motwani, 1998). Even with appropriate feature selection approaches, a large proportion of data is not utilized throughout the deep learning model training process, including patients without proper labeling or missing attributes. Therefore, approaches that impute missing values by their mean feature values before inference have been proposed (Beaulieu-Jones *et al.*, 2018; Fortuin *et al.*, 2020; Futoma *et al.*, 2017; Kang, 2013; Saunders *et al.*, 2006). However, they were reported to suffer from poor accuracy (ACC) for complicated underlying missing data distributions. A dedicated neural network was added to learn the underlying data distribution to achieve better performance (Wu *et al.*, 2020). Nonetheless, training complicated networks when data are scarce is prone to over-fitting. On the other hand, semi-supervised learning approaches were proposed such that the unlabeled data could be utilized jointly with labeled data throughout the training process for a better-performing model (Kingma *et al.*, 2014). Therefore, we could design model architectures to utilize all available data and achieve better overall performance without imputation.

To solve the issues mentioned above, we propose a robust Semi-supervised Cancer prognosis classifier with bAyesian variational autoeNcoder (*SCAN*) as a structured framework to utilize all available patient data. *SCAN* is trained in a semi-supervised manner and makes predictions based on the majority votes from all available data sources. Both censored patients and those with missing values can be utilized during training, alleviating the small data size issue and suppressing over-fitting.

We collected breast and NSCLC patient data cohorts to verify our model and built a prognosis prediction model based on their gene expression profiles and clinical information. The prognosis prediction targets are 5-year OS for NSCLC patients and DSS for breast cancer patients. Experiment results showed that *SCAN* achieved significantly better and more robust overall performance. One can apply *SCAN* to different cancer data; hence, it is a general framework for cancer prognosis prediction. Furthermore, *SCAN* is flexible, allowing new data sources to be incorporated throughout the training process. In general, our contribution is 3-fold:

1. We present a general framework to extract meaningful information from both labeled and unlabeled multimodal patient data by introducing *SCAN*. This allows researchers to fully utilize as much informative data as possible when building prediction models.
2. The proposed framework is lightweight and fast to train. We design the final prediction as the majority vote from all data modalities such that it is scalable when more data types are available. With an additional subnetwork classifier for a new data type, we may only need to re-adjust the weights for the votes.
3. We present *SCAN* as a robust semi-supervised framework that demonstrates its strengths in predicting the cancer prognosis of breast and NSCLC patients. Experiment results showed that (ensembled) *SCAN* achieved the best performance in most cases. We believe that *SCAN* can also generalize well to other cancer patient data.

## 2 Methods

### 2.1 Datasets and preprocessing

The patient data were collected from METABRIC (Curtis *et al.*, 2012; Pereira *et al.*, 2016) and Gene Expression Omnibus (GEO) repository for breast and NSCLC patients. To ensure a fair comparison, we kept the original test sets used in our previous works (Cheng *et al.*, 2021; Lai *et al.*, 2020), i.e. labeled patients were split into the training (80% for breast cancer; 66.7% for NSCLC) and test (20% for breast cancer; 33.3% for NSCLC) sets, respectively (Supplementary Section A). Following our previous works, with the help of well-known biomarkers, we utilized our systems biology feature selector to select a small set of prognostic biomarkers closely related to cancer prognosis prediction (Supplementary Section B). Based on our previous works (Cheng *et al.*, 2021; Lai *et al.*, 2020), these biomarkers were shown to deliver strong cancer prognosis prediction power with deep biological insights. Depending on whether the clinical data or survival labels are missing for the patients, we divided them into four types (Type I–IV) (Supplementary Section C). Type I patients have complete clinical data and survival labels. Type II patients have complete clinical information but without labels. Type III patients only have microarray data. Lastly, Type IV patients have missing values in their clinical information, but the corresponding labels can be defined. We summarize the key attribute distributions of the datasets in Table 1. Detailed patient data preprocessing steps can be found in Supplementary Section A.

**Table 1.** Patient attribute summary

	Labeled patients (Type I)					
	Breast cancer			NSCLC		
	Training	Validation	Test	Training	Validation	Test
# patients	349	116	117	256	85	171
Good prognosis	166 (47.56%)	55 (47.71%)	55 (47.01%)	177 (69.14%)	59 (69.41%)	119 (69.59%)
Poor prognosis	183 (52.42%)	61 (52.29%)	62 (52.99%)	79 (30.86%)	26 (30.59%)	52 (30.41%)
Median survival time (months)	57.23	54.43	56.27	59.08	55.1	49.6

	Unlabeled patients (Type II–IV)					

Type II	1168 (91.11%)	—	—	62 (60.78%)	—	—
Type III	114 (8.89%)	—	—	40 (39.22%)	—	—
Type IV	0 (0.00%)	—	—	0 (0.00%)	—	—

*Note*: Summary of patient attributes in the training, validation and test sets. We maintained the same test sets for both data cohorts as our original papers (Supplementary Section A). Patients with microarray data in the data cohorts were divided into four types (Supplementary Section C). No Type IV patients were identified in this study. Types II–IV unlabeled patients were used to assist semi-supervised model training.

We did not adopt traditional feature selection approaches for several reasons, and they were also discussed extensively in our previous works (Cheng *et al.*, 2021; Lai *et al.*, 2020). First of all, purely statistical feature selection approaches often fail to incorporate biological priors and end up with biomarkers with fewer biological insights. Secondly, most classic feature selection approaches are supervised that rely on prognosis survival labels, which are usually scarce in this case. Furthermore, they usually assign a distinct importance score for each gene independently of the rest, neglecting the complex underlying interaction between pairs of genes. Therefore, we developed our systems biology feature selector that selects prognostic biomarkers in an unsupervised fashion such that abundant unlabeled patient data can be fully utilized.

### 2.2 Study design

**Fig. 1.**
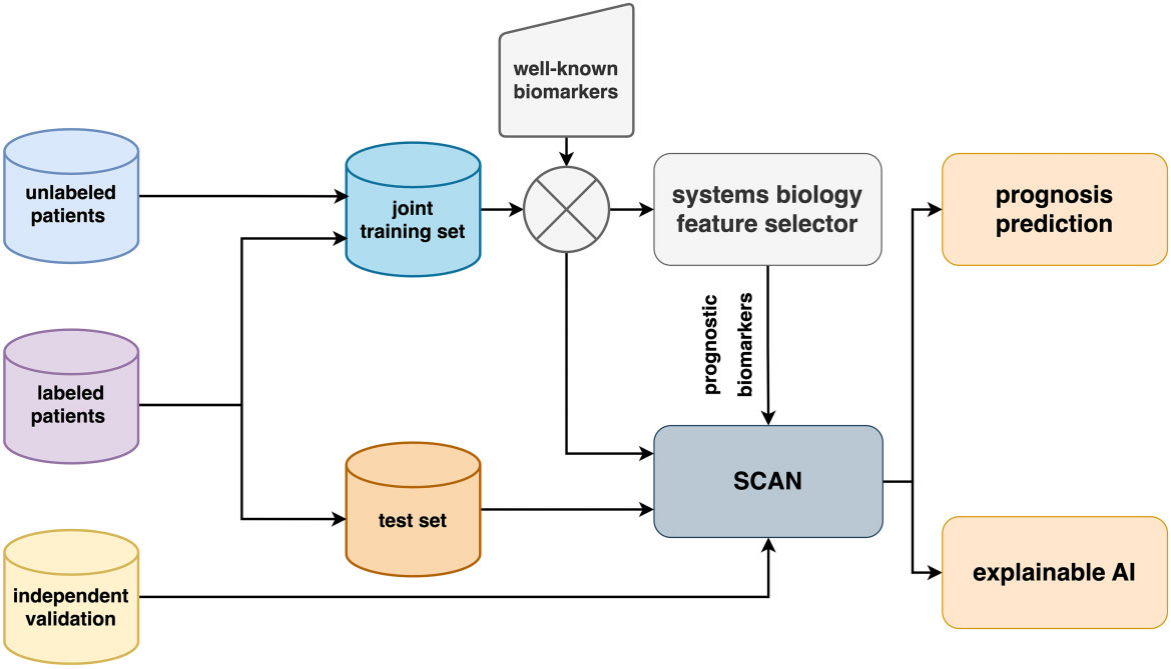
Overall framework. The labeled and unlabeled patient data formed the joint training and test set. Our systems biology feature selector selected prognostic biomarkers based on the well-known biomarkers from the joint training set. The models were trained on the joint training set with model hyper-parameters chosen via 4-CV. The test set and the independent validation set were then used to evaluate the model performance and generalizability. In addition to prognosis prediction, we further extract biological insights from the trained model through feature attribution analyses

We performed a retrospective study using microarray and clinical data from breast and NSCLC patients to predict their 5-year prognosis. The overall framework is illustrated in Figure 1. The joint training set combines the labeled training set and the data of unlabeled patients, including the ones with partially missing clinical information. The joint training sets were first fed into our systems biology feature selector to extract prognostic biomarkers with the help of the well-known biomarkers (Supplementary Section B). Four-fold cross-validations (4-CVs) on the joint training sets were then performed to select the hyper-parameters of *SCAN* (Supplementary Section D). In addition to the original test sets, we further included two independent validation datasets for each cancer to evaluate the robustness of the built models (Section 3.3). Based on the overall framework shown in Figure 1, we presented two case studies to validate *SCAN*, including the prediction of the 5-year OS of NSCLC patients as well as predicting the 5-year DSS for breast cancer patients. Specifically, we selected a set of 20 and 15 prognostic biomarkers for breast and NSCLC, respectively (Supplementary Section A). We further included the available clinical information during training.

### 2.3 Robust *SCAN*

**Fig. 2.**
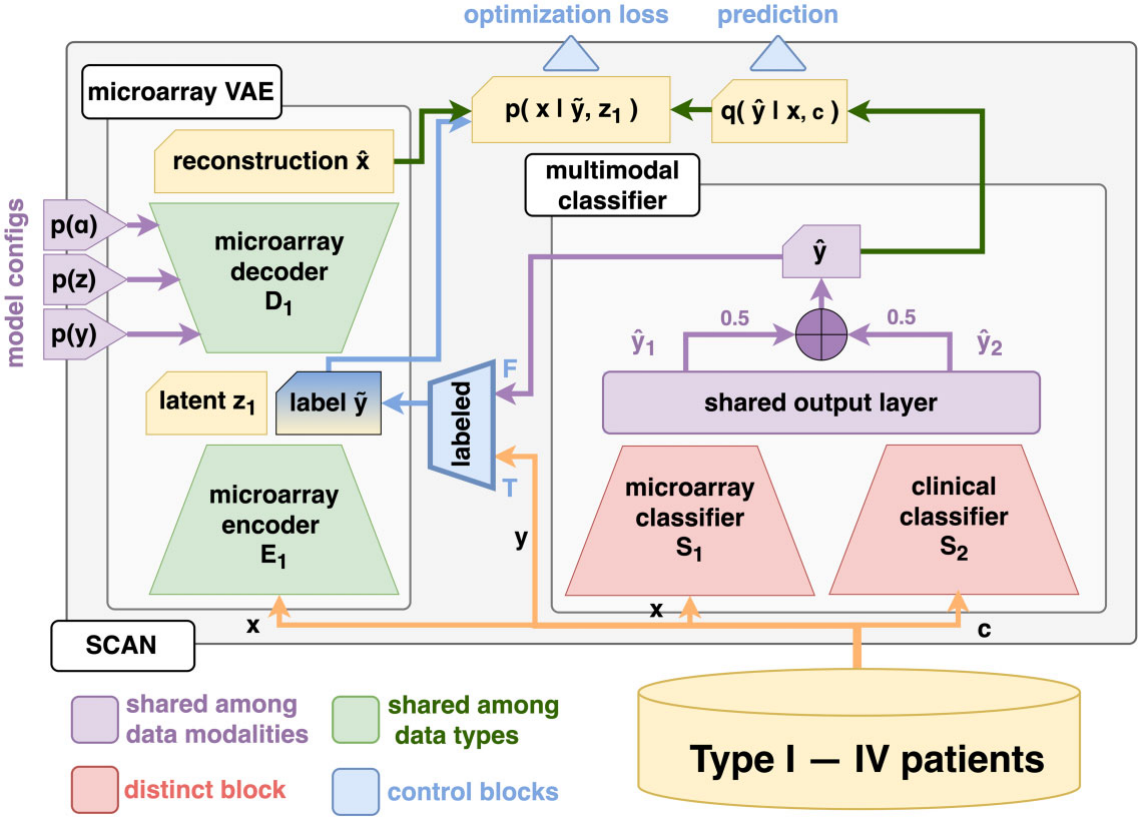
Robust *SCAN*. The proposed model consists of a microarray VAE and a multimodal classifier. The microarray VAE learns low-dimensional gene profile representation and assists in using unlabeled patient data for semi-supervised learning. There are two distinct classifiers for microarray and clinical patient data, respectively. The shared output layer weighted the temporary predictions for the two classifiers ($\hat{y_{1}},\;\hat{y_{2}}$). Finally, equally weighted votes from the two classifiers made the final prediction in an ensemble fashion ($\tilde{y}$). Dedicated loss functions were designed for different types of patients, and the model was trained with back-propagation. The purple blocks are shared among subnetworks, including the shared output layer and model prior probability configurations. Detailed settings of the priors can be found in Supplementary Section D. The green blocks (the VAE) are shared among distinct data types (microarray and clinical data). The red blocks are designated for each data type, such as the microarray and clinical subnetwork classifiers. The blue blocks represent semi-supervised learning-related algorithms (Supplementary Section C)

We proposed *SCAN* as a semi-supervised learning framework that can incorporate all heterogeneous patient data (potentially with missing values) from various sources. The basic component of *SCAN* borrows ideas from a semi-supervised learning variational autoencoder (SSL-VAE) block that can take advantage of both labeled and unlabeled data to learn a powerful classifier, as detailed by Kingma *et al.* (2014). Their original work focused only on image generation tasks with a single data modality. We generalize the model to accept multimodal inputs such that the proposed model is scalable, robust and feasible for federated learning (Section 4). In the current application scenario, two data sources (or data types, namely, microarray and clinical data) are available, and an SSL-VAE block can be deployed for each data type. The likelihood function of each data is modeled with its corresponding VAE, which is updated throughout the training process. However, because we have fewer clinical features than those for microarray in the collected cohorts (especially in the NSCLC cohort), we observed a smoother training process and reduced risk of over-fitting for the clinical data without the clinical VAE. As a result, the resulting architecture of *SCAN* used in this work consists of a microarray VAE and classifier, whereas only a classifier was trained for the clinical data. If much richer clinical data were given for the NSCLC cohort, we could explore different model architectures as one of the future improvements. The overall model architecture is illustrated in Figure 2.

We implemented a full variational Bayes VAE (Kingma and Welling, 2013) with variational dropout (Kingma *et al.*, 2015) and applied L2-regularization (Hastie, 2020) on neural network weights to combat over-fitting. More model details, such as the objective function formulations of *SCAN*, can be found in Supplementary Section C. A shared output layer was inserted before generating the final prediction. This shared layer also allows us to merge more than two data sources for future applications. *SCAN* can adjust the importance of subnetwork predictions (‘votes’) through the learnable weights in the shared layer. The weighted votes are then combined with equal importance for the final prediction. Because of its ensemble nature, *SCAN* can generate predictions with all kinds of data modalities (microarray only, clinical only or both) with or without labels. All of the continuous data were standardized during training to alleviate numerical instability caused by large gradients. *SCAN* can be potentially applied to even more data modalities if such data are available.

### 2.4 Benchmark models and performance evaluation metrics

We compared the performance of *SCAN* with three benchmark models, including the bimodal neural network classifier proposed in our previous papers (*Bimodal*) (Cheng *et al.*, 2021; Lai *et al.*, 2020), support vector machine (SVM) (Chang and Lin, 2011) and random forest (RF) (Breiman, 2001) classifiers with five famous evaluation metrics, i.e. the area under the receiver operating curve (AUROC) (Fawcett, 2006), macro *F*1-score (macro *F*1) (Powers, 2020), concordance index (CI) (Harrell *et al.*, 1996), ACC and area under the precision-recall curve (AUPRC) (Zhu, 2004). The performance metrics were accompanied by 95% confidence intervals observed from 1000 bootstrap test sets (Supplementary Section E). The main performance evaluation metrics are AUROC (Fawcett, 2006) and CI (Harrell *et al.*, 1996). In addition, we performed extensive analyses to confirm the robustness of the built models (Section 4) and extract the underlying biological insights learned from the data (Supplementary Sections G–I).

## 3 Results

### 3.1. Case study: breast cancer 5-year DSS prediction with *SCAN*

In the first case study, we applied *SCAN* to predict breast cancer prognosis. We identified 1902 patients with microarray profiles, and 582 of them have microarray expressions, clinical features and appropriately defined labels from METABRIC (Curtis *et al.*, 2012; Pereira *et al.*, 2016). Based on our previous research (Cheng *et al.*, 2021), 20 prognostic biomarkers (ESR1, PGR, ERBB2, MKI67, PLAU, ELAVL1, EGFR, BTRC, FBXO6, SHMT2, KRAS, SRPK2, YWHAQ, PDHA1, EWSR1, ZDHHC17, ENO1, DBN1, PLK1 and GSK3B) (Supplementary Section A) and 10 clinical features (age, menopausal state, tumor size, radiotherapy, chemotherapy, hormone therapy, neoplasm histologic grade, cellularity, surgery-breast conserving and surgery-mastectomy) were included in the experiments. Labeled patient data were utilized for training the microarray VAE and both subnetworks (microarray and clinical), while unlabeled ones helped in learning the microarray VAE (Supplementary Section C).

**Fig. 3.**
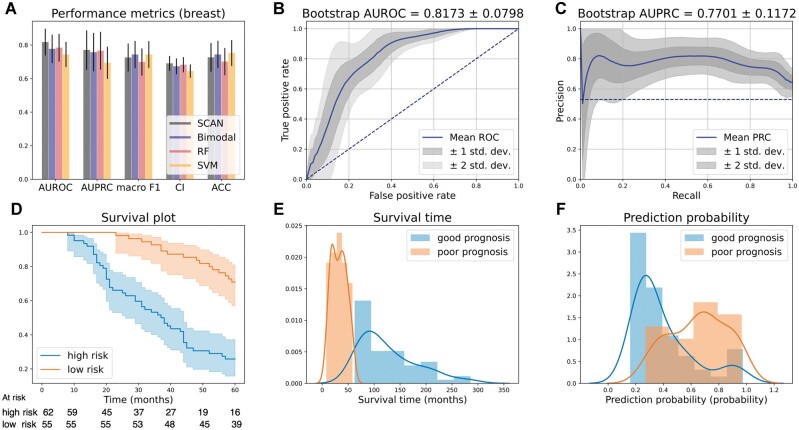
Breast cancer DSS. (**A**) Model performance comparison. The bars represent the test set performance, and the black error bars represent the corresponding 95% confidence intervals estimated with 1000 bootstrap test sets. (**B**) ROC curves. The blue curve is the mean curve averaged over 1000 ROC curves from the bootstrap test sets. Precision scores were calculated at 100 equally spaced false-positive rates in [0, 1], and the variation of the ROC curves was estimated. (**C**) PRC. The blue curve is the mean curve averaged over 1000 PRC curves from the bootstrap test sets. Precision scores were calculated at 100 equally spaced recall rates in [0, 1], and the variation of the PRC curves was estimated. (**D**) Survival analysis. The KM plot was plotted for predicted good and poor prognosis patients. The log-rank test showed significant stratification (*P* < 0.0001). (**E**) Survival time stratification. The patients were divided into good and poor prognosis classes, and the distribution of the survival time of the two groups is illustrated. (**F**) Predicted event probability

Model performance is summarized in Table 2. Results for breast cancer prognosis prediction are illustrated in Figure 3. As shown in Figure 3A, *SCAN* (81.73%) performed the best in AUROC among all models, with an approximate 4% improvement gained from the unlabeled data over *Bimodal* (77.71%). *SCAN* also achieved the best CI (69.02%) compared to the other models, which means that *SCAN* could identify the patients with short survival time with high-risk scores. Ablation studies showed that most performance metrics dropped significantly by gradually removing the loss for Type III to Type I patients (Supplementary Section F). The AUROC dropped from 81.73% (*SCAN*) to 79.68% when Types III and II patients were excluded from training. The performance further dropped to 77.74% when the microarray VAE for Type I patients was removed. The performance worsened when less unlabeled patient data were available (Section 4). We then concluded that the performance gains observed in AUROC and CI stemmed from the larger unlabeled patient data.

**Table 2.** Performance summary for predicting 5-year DSS for breast cancer patients

Metrics (%)	*SCAN*	*Bimodal*	RF	SVM
AUROC	**81.73 (7.98)**	77.71 (8.46)	78.36 (8.25)	74.35 (7.50)
CI	**69.02 (4.43)**	67.33 (4.68)	68.28 (4.55)	64.45 (3.95)
macro *F*1	72.55 (8.33)	**74.34 (8.06)**	69.87 (8.08)	74.31 (8.02)
AUPRC	**77.01 (11.72)**	75.71 (11.44)	76.61 (11.23)	69.35 (9.56)
ACC	72.65 (8.55)	74.36 (8.12)	70.09 (8.12)	**75.21 (7.69)**

*Note*: *SCAN* achieved the best AUROC and CI among all models while achieving comparable performance with the best models for the other metrics. Summary of performance metrics (testing on the original cohorts) and the corresponding 95% confidence intervals from 1000 bootstrap test sets are shown in parentheses. Best-performing cases are bold-faced.

The receiver operating characteristics curves (ROC curves) and precision-recall curves (PRCs) with one and two standard deviation(s) in 1000 bootstrapped test sets are shown in Figure 3B and C, respectively. The dashed lines represent random guesses. We observed a more concentrated ROC curves distribution than PRCs, which may be strongly affected by the class imbalance (poor-to-good) ratios among 1000 bootstrap test sets. This observation is in accordance with the fact that PRCs are more sensitive to label imbalance than ROC curves (Saito and Rehmsmeier, 2015), and thus, a more unstable behavior is expected. The survival plot in Figure 3D shows significant stratification of the predicted poor and good prognosis subgroups (log-rank *P* < 0.0001). In Figure 3E, the patient survival time in each predicted subgroup (poor/good prognosis) was plotted. We observed a short survival time of 32.33 months (median) for patients in the predicted high-risk group. In contrast, the ones predicted as low-risk had a rather long survival time (median: 110.83 months). Figure 3F shows that high-risk patients’ predicted DSS event probabilities have a different distribution than the low-risk ones. In addition, we found that the learned latent representation from the VAE can potentially infer patient prognosis information (Supplementary Section G). We identified the most important features learned from the classifier with the connection weights algorithm (Olden and Jackson, 2002) (Supplementary Section H) and partial dependency plots (Goldstein *et al.*, 2015) (Supplementary Section I) to infer biological insights from the data. Among all selected prognostic biomarkers, ESR1, PGR, BRTC, YWHAQ and PLK1 were recognized as crucial features; for clinical features, whether the patient has gone through chemotherapy/hormone therapy and the size of the tumor play an important role in model predictions.

### 3.2. Case study: NSCLC 5-year OS prediction with *SCAN*

For NSCLC, we collected 614 patients, and 512 of them have full microarray profiles, clinical data and labels. We used our systems biology feature selector (Supplementary Section B) to select 15 prognostic biomarkers (EPCAM, HIF1A, PKM, PTK7, ALCAM, CADM1, SLC2A1, CUL1, CUL3, EGFR, ELAVL1, GRB2, NRF1, RNF2 and RPA2). Three clinical features (age, gender and stage) were left after intersecting six GEO datasets (Lai *et al.*, 2020).

**Fig. 4.**
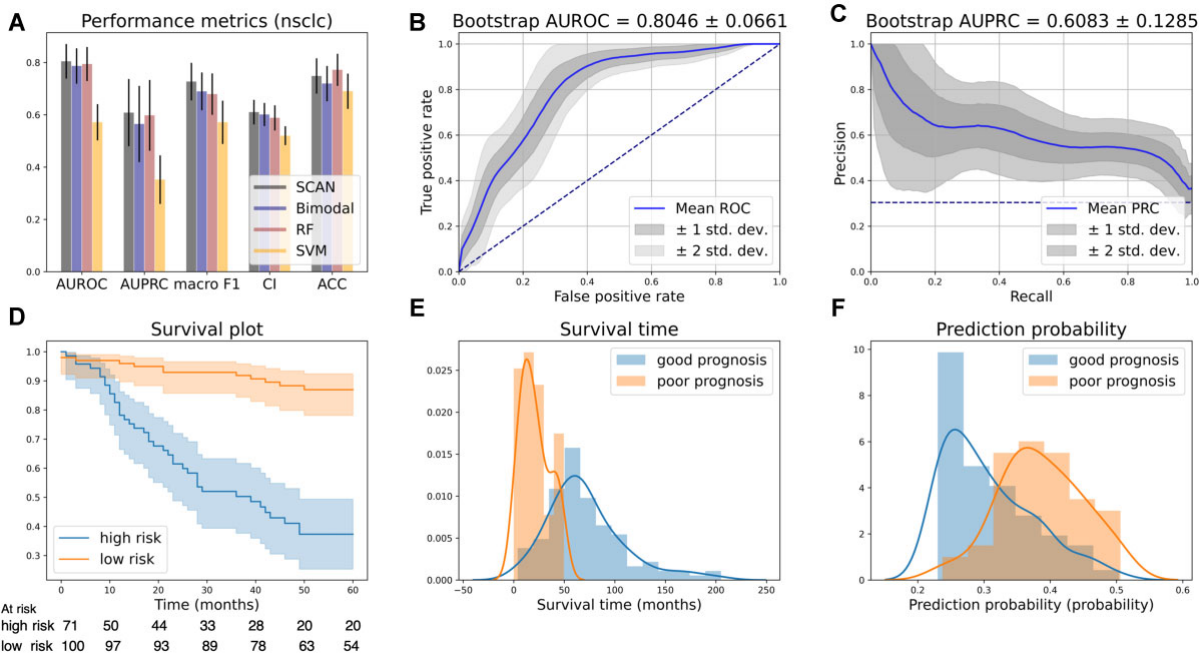
NSCLC OS. (**A**) Model performance comparison. The bars represent the test set performance, and the black error bars represent the corresponding 95% confidence intervals estimated with 1000 bootstrap test sets. (**B**) ROC curves. The blue curve is the mean curve averaged over 1000 ROC curves from the bootstrap test sets. Precision scores were calculated at 100 equally spaced false-positive rates in [0, 1], and the variation of the ROC curves was estimated. (**C**) PRC. The blue curve is the mean curve averaged over 1000 PRC curves from the bootstrap test sets. Precision scores were calculated at 100 equally spaced recall rates in [0, 1], and the variation of the PRC curves was estimated. (**D**) Survival analysis. The KM plot was plotted for predicted good and poor prognosis patients. The log-rank test showed significant stratification (*P* < 0.0001). (**E**) Survival time stratification. The patients were divided into good and poor prognosis classes, and the distribution of the survival time of the two groups is illustrated. (**F**) Predicted event probability

The performance is summarized in Table 3 and Figure 4. As shown in Figure 4A, *SCAN* performed the best among all models in all metrics except for ACC. Furthermore, *SCAN* (AUROC: 80.46%; macro *F*1: 72.70%; CI: 61.03%; AUPRC: 60.83%; ACC: 74.85%) outperformed *Bimodal* (AUROC: 78.67%; macro *F*1: 68.95%; CI: 60.12%; AUPRC: 56.50%; ACC: 71.93%) in all metrics, with approximately 2%, 3%, 1%, 4% and 2% superior to *Bimodal* in AUROC, macro *F*1, CI, AUPRC and ACC, respectively. To evaluate the robustness of predictions, we again plotted the bootstrapped ROC curves and PRCs as illustrated in Figure 4B and C, respectively. Similarly, we observed that the ROC curves distribution was more concentrated than PRCs’ because the NSCLC cohort suffers from a more severe label imbalance issue than the breast cancer cohort. As illustrated in Figure 4D, the Kaplan–Meier (KM) plot shows that *SCAN* can provide strong stratification between poor and good prognosis patients (log-rank *P* < 0.0001). Figure 4E and F shows that the predicted low-risk patient subgroup enjoys longer survival times (median: 63.60 months for low-risk patients; 18.27 months for high-risk ones). Further analyses on the learned VAE latent representation (Supplementary Section G) and how to identify crucial features for model risk prediction (Supplementary Sections H and I) can be found in Supplementary Material. From our analysis, CADM1, SLC2A1, ELAVL1, NRF1 and the stage were considered the most important features for prognosis risk prediction.

**Table 3.** Performance summary for predicting 5-year OS for NSCLC patients

Metrics (%)	*SCAN*	*Bimodal*	RF	SVM
AUROC	**80**.**46 (6**.**61)**	78.67 (6.75)	79.41 (6.51)	57.16 (6.93)
CI	**61**.**03 (4**.**70)**	60.12 (4.40)	58.82 (4.80)	52.01 (3.60)
macro *F*1	**72**.**70 (7**.**18)**	68.95 (7.24)	67.90 (7.92)	57.13 (8.27)
AUPRC	**60**.**83 (12**.**85)**	56.50 (14.55)	59.79 (13.51)	35.22 (9.26)
ACC	74.85 (6.73)	71.93 (6.73)	**77**.**19 (6**.**14)**	69.01 (6.73)

*Note*: *SCAN* performed the best among all metrics except for ACC. Summary of performance metrics (testing on the original cohorts) and the corresponding 95% confidence intervals from 1000 bootstrap test sets are shown in parentheses. Best-performing cases are bold-faced.

### 3.3. External validation

To evaluate the robustness of the model, we used an additional external validation dataset for each cancer: E-MTAB-923 (*n* = 91) for NSCLC and GSE21653 (*n* = 83) for breast cancer. However, since specific clinical features included in METABRIC are missing from GSE21653, we focused instead on predicting the 5-year disease-free survival with only microarray expression profiles. Interested readers may resort to Supplementary Section J in Cheng *et al.* (2021) for more detailed discussions. In that work, Cheng *et al.* did not use GSE21653 to identify the biomarkers, and thus it is appropriate to use it as an external validation dataset. We tested *SCAN* and *Bimodal* trained with the previous joint training set on these two external validation datasets. Furthermore, we also included the ensemble version of both models in comparison. For model ensembles, 200 models with identical hyper-parameters chosen with 4-CV (Supplementary Section D) but with different random seeds were first trained. The final prediction was generated by averaging the 200 logits from all models. The results are summarized in Table 4. In summary, *SCAN*-based models achieved robust predictions in AUROC, AUPRC and CI. In contrast, *Bimodal*-based models suffered from more severe over-fitting that prevented them from generalizing to unseen external validation datasets.

**Table 4.** External validation for breast and NSCLC prognosis predictions

Breast cancer				
Metrics (%)	*Ensemble SCAN*	*SCAN*	*Ensemble Bimodal*	*Bimodal*
AUROC	**75.67 (13.69)**	74.74 (13.39)	73.29 (14.34)	64.13 (17.08)
CI	**65.68 (5.89)**	65.47 (5.83)	64.17 (6.08)	61.61 (6.22)
macro *F*1	58.56 (11.36)	42.54 (10.44)	**63.52 (13.10)**	45.39 (2.35)
AUPRC	93.36 (5.93)	**93.62 (5.21)**	93.08 (5.67)	88.26 (8.82)
ACC	68.67 (10.24)	43.37 (10.26)	80.72 (8.43)	**83.13 (7.83)**

Non-small cell lung cancer				

Metrics (%)	*Ensemble SCAN*	*SCAN*	*Ensemble Bimodal*	*Bimodal*

AUROC	71.64 (11.28)	**72.80 (11.24)**	54.40 (12.09)	67.07 (11.30)
CI	**59.27 (7.33)**	58.87 (7.16)	48.13 (7.36)	54.07 (6.67)
macro *F*1	**55.88 (10.76)**	41.57 (7.71)	36.17 (4.09)	36.17 (4.09)
AUPRC	68.69 (14.14)	**69.75 (13.87)**	46.46 (14.66)	58.54 (15.27)
ACC	**61.11 (10.00)**	58.89 (10.00)	56.67 (10.00)	56.67 (10.00)

*Note*: *SCAN*-based models performed better than *Bimodal*-based models. The summary of performance metrics (testing on the original cohorts) and the corresponding 95% confidence intervals from 1000 bootstrap test sets are shown in parentheses. Best-performing cases are bold-faced.

For breast cancer, *SCAN* (74.74%) achieved better AUROC than *Bimodal* (64.13%). *SCAN* (65.47%) also outperformed *Bimodal* (61.61%) in terms of CI. We can observe similar results in the case of *Ensemble SCAN* and *Ensemble Bimodal*. As for NSCLC, the best AUROC score was achieved by *SCAN* (72.80%), while *Bimodal* (67.07%) achieved a much inferior prediction result. *Ensemble SCAN* (59.27%) achieved the best CI, while *Ensemble Bimodal* performed the worst (48.13%). Due to the discrepancy between the joint training set from the original cohorts and the external validation datasets, we observed a performance drop and larger confidence intervals for both cancers when testing on the corresponding independent validation datasets. However, even with such different patient feature distributions and weight initializations (200 random seeds), we still observed that *(Ensemble) SCAN* yields decent prediction results. These observations conclude that *SCAN* becomes much more robust with the introduction of large unlabeled patient data.

### 3.4. Validation on the TCGA datasets

We also tested *SCAN* on The Cancer Genome Atlas (TCGA) datasets as another external validation. In particular, we collected patient data from TCGA-BRCA and TCGA-LUAD as another external validation dataset for breast and NSCLC. The results are summarized in Supplementary Section J. We observed that the microarray subnetwork classifier suffered from performance degradation. We suggested that this was mainly due to the drastic different distribution between microarray and RNA-Seq data. They use different technologies with different normalization methods. In addition, we observed that the label imbalance ratios (i.e. the ratio of the numbers of patients from majority to minority class) are different (microarray: 62/55 = 1.13 for breast cancer and 119/52 = 2.29 for NSCLC; RNA-Seq: 119/20 = 5.95 for TCGA-BRCA and 68/34 = 2.00 for TCGA-LUAD). These complications all contributed to the performance degradation. However, the clinical features still contribute enough to the prediction. As a result, based on the majority vote design adopted in *SCAN*, it still yields decent overall performance.

## 4 Discussion

### 4.1. *SCAN* is robust to model initialization

It was reported that only subtle network parameter initialization changes the resulting prediction by a comparable amount, especially in deep networks (Dusenberry *et al.*, 2020). In deep learning literature, one may choose the best hyper-parameter based on the initialization of one specific random seed (and thus with a particular weight initialization). This significantly undermines the applicability of deep learning in practical scenarios. As a result, it is more convincing to provide network predictions averaged over many random seeds to ensure that the model does not generate good predictions by coincidence (Bishop, 2006; Dusenberry *et al.*, 2020; Lakshminarayanan *et al.*, 2017). This approach is also often adopted in machine learning-related research where the robustness of the feature selection is measured by the expected value and variance of the predictions over the model ensemble (Lakshminarayanan *et al.*, 2017). Therefore, we trained 200 *SCAN* and *Bimodal* networks with identical hyper-parameters chosen with 4-CV (Supplementary Section D) but with 200 different random seeds, respectively. We then summarized the averaged prediction performance among 200 models in the ensemble and measured the prediction’s variance with a 95% confidence interval in 1000 bootstrap test sets. The final prediction is the average of over 200 predictions in the model ensemble. For such model ensembles, the results tested on the original cohorts are summarized in Table 5. Observations showed that Ensemble *SCAN* achieved superior performance for breast and NSCLC patients than Ensemble *Bimodal* in almost all metrics except AUPRC. The widths of the 95% CIs were approximately the same. For breast cancer patients, results showed that Ensemble *SCAN* performed the best by achieving the best AUROC (75.67%) compared to *SCAN* (74.74%), *Bimodal* (64.13%) and Ensemble *Bimodal* (73.29%). The ensemble models performed much better than their non-ensembled counterparts. As for NSCLC, we showed that *SCAN* (AUROC: 72.80%; AUPRC: 69.75%) performed slightly better than Ensemble *SCAN* (AUROC: 71.64%; AUPRC: 68.69%) in AUROC and AUPRC scores but worse for the other metrics. *Bimodal* (AUROC: 67.07%; AUPRC: 58.54%) and Ensemble *Bimodal* (AUROC: 54.40%; AUPRC: 46.46%) had really poor performance. We suspected Ensemble Bimodal performed worse than Bimodal because *Bimodal* might generate different predictions sensitive to different random seeds for weight initialization. On the other hand, Ensemble *SCAN* showed strong robustness against different model initialization.

**Table 5.** Performance summary for Ensembled version of *SCAN* and *Bimodal*

Metrics (%)	Breast *SCAN*	Breast *Bimodal*	NSCLC *SCAN*	NSCLC *Bimodal*
AUROC	**80**.**65 (8**.**32)**	78.91 (8.38)	**80**.**32 (6**.**68)**	78.62 (6.96)
macro *F*1	**75**.**55 (7**.**56)**	70.01 (8.14)	**72**.**32 (7**.**45)**	41.03 (2.35)
CI	**68**.**79 (4**.**52)**	68.38 (4.56)	**61**.**20 (4**.**62)**	60.00 (4.34)
AUPRC	75.45 (11.82)	**77**.**47 (10**.**95)**	60.12 (13.22)	**62**.**85 (11**.**45)**
ACC	**76**.**07 (7**.**28)**	70.09 (8.12)	**73**.**68 (7**.**02)**	69.59 (6.73)

*Note*: We observed that Ensemble *SCAN* outperformed Ensemble *Bimodal* in all metrics but with less AUPRC. The summary of performance metrics (testing on the original cohorts) and the corresponding 95% confidence intervals from 1000 bootstrap test sets are shown in parentheses. Best-performing cases are bold-faced.

### 4.2. *SCAN* improves with increasing unlabeled data

**Fig. 5.**
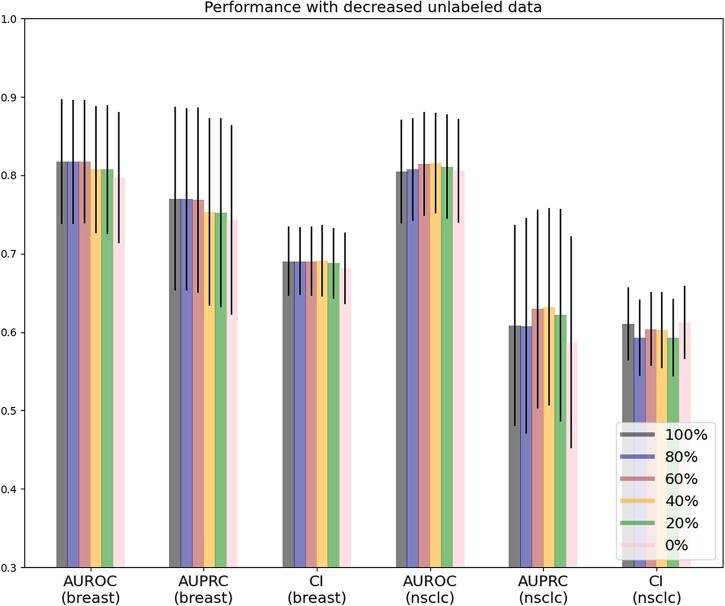
Prognosis prediction with decreased unlabeled data. *SCAN*’s performance dropped with less unlabeled data, which was more prominent for breast cancer

To investigate the contribution of unlabeled data in improving performance, we conducted another numerical experiment similar to the ablation study in the previous section. We deliberately removed some proportion of the unlabeled data from the training set while maintaining the overall loss function. The models were trained with only 20%, 40%, 60% and 80% of the original unlabeled data in the training set. We fixed the hyper-parameters chosen from 4-CV for all cases. The results are summarized in Figure 5. Experimental results showed that when the number of unlabeled data increased from 20% to 100%, the resulting performance improved significantly. Due to different unlabeled patient sample sizes, we observed a more noticeable trend for breast cancer than among NSCLC patients. As a result, we expect the prediction performance to improve further when more unlabeled data are available (more than 100%). However, since data collection for large unlabeled patient data is not easy, to verify our assumption, we augmented the joint training set with more unlabeled data by simply duplicating the original ones. We can, therefore, arbitrarily increase the amount of unlabeled patient data but restrict it to the identical amount of information provided by the original ones. As we tried to include even more unlabeled data, *SCAN* improved initially when the unlabeled data were doubled (Supplementary Section K). The performance began to drop when more unlabeled data were duplicated. The duplication process might initially introduce certain uncertainty/noise into the training process. However, model performance could not improve indefinitely even with synthetic data generated with advanced generative adversarial network (GAN) (Jahanian *et al.*, 2021; Wei *et al.*, 2022). As a result, we plan to search for larger and more consistent data cohorts, such as TCGA, for more thorough analysis as a future research direction. Potential future works are summarized in Supplementary Section L.

## 5 Conclusion

In this work, we proposed *SCAN* that supports semi-supervised learning. Precious patient data could be utilized fully to train a powerful model under a unified framework. Detailed analyses showed that *SCAN* (AUROC: 81.73%, CI: 69.02% for breast cancer; AUROC: 80.46%, CI: 61.03% for NSCLC) achieved better and more robust performance compared to other models (AUROC: 77.71%, CI: 67.33% for breast cancer with *Bimodal*; AUROC: 78.67%, CI: 60.12% for NSCLC with *Bimodal*). *SCAN* can provide even better performance as the number of unlabeled patients increases, and it can be easily scaled to include more heterogeneous data types. The training time of *SCAN* is short, and one can expand it to an ensemble version, which again provides the best performance among all variations. This paves the foundation of personalized medicine that various types of (even unlabeled) patient data can be fully utilized to train a powerful unified model under a federated learning framework. To further verify the generalizability of *SCAN*, we need to train the framework on even larger patient cohorts with more patients and more abundant clinical features, such as TCGA. We also plan to re-design the microarray subnetwork in *SCAN* for RNA-Seq data based on next-generation sequencing (Tomczak *et al.*, 2015). We will need to revisit our feature selection processes for selecting appropriate new biomarkers (Wu *et al.*, 2019; Zhao *et al.*, 2015). These are set as our future works.

## Supplementary Material

vbac100_Supplementary_Data

## Data Availability

The gene expression profiles and clinical information are all freely available on the Gene Expression Omnibus (GEO) repository at National Center for Biotechnology Information (NCBI) (https://www.ncbi.nlm.nih.gov/geo) for NSCLC patients (GSE19188, GSE29013, GSE30219, GSE31210, GSE37745, GSE50081). The METABRIC datasets were used for breast cancer patients and can be downloaded from cBioPortal, an online breast cancer cohort.
