# Supplementary material for: Learning from small medical data—robust semi-supervised cancer prognosis classifier with Bayesian variational autoencoder
Source: Bioinform Adv. 2023 Jan 9;3(1):vbac100. doi: 10.1093/bioadv/vbac100 (PMC9832968; doi:10.1093/bioadv/vbac100)
Supplement: vbac100_Supplementary_Data [file vbac100_supplementary_data.docx]

**Supplementary Materials**

**Learning from Small Medical Data - Robust Semi-supervised Cancer Prognosis Classifier with Bayesian Variational Autoencoder**

Te-Cheng Hsu^1^ & Che Lin^2†^

^1^Institute of Communications Engineering, National Tsing Hua University, Hsinchu 30013, R.O.C. (Taiwan)

^2†^Department of Electrical Engineering & Graduate Institute of Communication Engineering, National Taiwan University, Taipei 10617, R.O.C. (Taiwan)

^†^: corresponding author

**A. Data preprocessing and data distribution**

Breast cancer patient data were extracted from the METABRIC^1,2^ dataset, and patient data of NSCLC were collected from six GEO datasets, including GSE19188, GSE29013, GSE30219, GSE31210, GSE37745, GSE50081. We followed the same data preprocessing procedures in our previous research^3,4^. The test sets were kept the same while the training set was divided into four splits for four-fold cross-validation (4-CV). The identical sets of prognostic biomarkers selected from the systems biology feature selector were used (*Supplementary B*). The essential clinical feature distributions of the labeled patients for breast and non-small cell lung cancer patients were summarized in Tables S1 in Cheng *et al.*^4^*,* and Table S1 in the paper by Lai *et al.*^3^*,* respectively. Here, we refer patients without properly assigned labels (5-year OS/DFS) as unlabeled patients, and they may have missing values in the microarray and clinical data. For METABRIC, 1,902 patients have microarray profiles, and 582 have complete microarray, clinical, and label information. The patients were then split into the training (n = 465) and test (n = 117) sets. Among the rest, 1,282 unlabeled patients with microarray profiles, only 114 of them have complete clinical data (compared to those 582 labeled patients), and 1,168 of them are left without complete clinical data.

On the other hand, for NSCLC, there are 614 patients in the collected cohort, and 512 of them are labeled (n = 341 for the training set; n = 171 for the test set). The rest 102 unlabeled patients are without complete clinical data. The unlabeled data were normalized according to the training set distributions. They were all utilized to select prognostic biomarkers and joined the training process in a semi-supervised fashion, as detailed in *Supplementary C*.

**Table S1. Prognostic biomarkers.** The prognostic biomarker sets used in this work were selected with our systems biology feature selector (*Supplementary B*), and well-known biomarkers are bold-faced.

| **Breast cancer** | **ESR1**, **PGR**, **ERBB2**, **MKI67**, **PLAU**, ELAVL1, EGFR, BTRC, FBXO6, SHMT2, KRAS, SRPK2, YWHAQ, PDHA1, EWSR1, ZDHHC17, ENO1, DBN1, PLK1, GSK3B |
| --- | --- |
| **NSCLC** | **EPCAM**, **HIF1A**, **PKM**, **PTK7**, **ALCAM**, **CADM1**, **SLC2A1**, CUL1, CUL3, EGFR, ELAVL1, GRB2, NRF1, RNF2, RPA2 |

**B. Systems biology feature selector**

We adopted the biomarkers selected from our previous researches^3,4^ according to our systems biology feature selector that infers important biological insights through gene interaction networks (GINs). We first adopt several well-known biomarkers as the split criteria and then build several pairs of GINs based on the gene expression profiles. Candidate genes are ranked by their corresponding prognostic relevance values (PRVs) calculated from built GINs. Top-scoring genes and well-known biomarkers are collected as the final prognostic biomarkers used to train our model. For the rest of the section, we will go through every step of the systems biology feature selector.

The patients were divided into two groups according to the binary split criteria assigned. The split criteria are high versus low well-known biomarker expression subgroups stratified with StepMiner algorithm^5^. Genes without significant separation between the two groups were excluded by the analysis of variance (ANOVA) algorithm. Based on the selected patients and genes, we constructed candidate gene interaction networks (GINs) for each group based on the interaction information from the BioGRID database^6^. Selected gene expression profiles were then utilized to estimate the interaction ability between genes, and false-positive interactions were removed via Akaike’s information criterion (AIC) and Student’s t-test. The constructed GINs would therefore be tailored for specific cancer in consideration.

We assume that the expression level of a gene is contributed by the other interacting genes through a linear combination of their expression levels:

| $x_{i}\left[ n \right]=\sum_{j\in G_{i}} a_{ij}x_{j}\left[ n \right]+\varepsilon_{i}\left[ n \right],$ | (1) |
| --- | --- |

where $x_{i}\left[ n \right]$ is the expression level of gene $i$ for patient $n$; $a_{ij}$ is the interaction ability between genes $i$ and $j$; $G_{i}$ is the set of genes that are related to gene $i$ in BioGRID; and $\varepsilon_{i}\left[ n \right]$ is stochastic noise. Stacking equation (1) for every selected patient and gene, we have:

| $\boldsymbol{X}=\boldsymbol{AX}+\boldsymbol{E,}$ | (2) |
| --- | --- |

where

$\boldsymbol{X}=\left[ \begin{matrix} x_{1}\left[ 1 \right] & \cdots& x_{1}\left[ N \right] \\ \vdots& \ddots& \vdots\\ x_{M}\left[ 1 \right] & \cdots& x_{M}\left[ N \right] \end{matrix} \right]$**,** $\boldsymbol{A}=\left[ \begin{matrix} a_{11} & \cdots& a_{1M} \\ \vdots& a_{ij} & \vdots\\ a_{M1} & \cdots& a_{MM} \end{matrix} \right]$, $\boldsymbol{E}=\left[ \begin{matrix} \varepsilon_{1}\left[ 1 \right] & \cdots& \varepsilon_{1}\left[ N \right] \\ \vdots& \ddots& \vdots\\ \varepsilon_{M}\left[ 1 \right] & \cdots& \varepsilon_{M}\left[ N \right] \end{matrix} \right].$

where *M* is the number of genes after ANOVA, and *N* is the number of patients in a subgroup. The interaction abilities were obtained by solving the Linear Minimum Square Error (LMMSE) problems for each patient subgroup. False-positive interactions were then removed with AIC and t-test. If $a_{ij}$ is not equal to $a_{ji}$, we took the one with the larger absolute value as the final interaction ability. GINs from the positive and negative patient subgroup are denoted as A^+^ and A^-^, respectively. We further define the difference matrix D to be:

| $\boldsymbol{D}=\boldsymbol{A}^{+}-\boldsymbol{A}^{-}=\left[ \begin{matrix} d_{11} & \cdots& d_{1M} \\ \vdots& \ddots& \vdots\\ d_{M1} & \cdots& d_{MM} \end{matrix} \right]=\left[ \begin{matrix} a_{11}^{+}-a_{11}^{-} & \cdots& a_{1M}^{+}-a_{1M}^{-} \\ \vdots& \ddots& \vdots\\ a_{M1}^{+}-a_{M1}^{-} & \cdots& a_{MM}^{+}-a_{MM}^{-} \end{matrix} \right],$ | (3) |
| --- | --- |

where $d_{ij}$ is the difference in the interaction ability between genes $i$ and $j$. The prognosis relevance value (PRV) is then defined as:

| ${PRV}_{i}=\sum_{j=1}^{M} \left\vert d_{ij} \right\vert,$ | (4) |
| --- | --- |

which is the summarized interaction ability differences between gene $i$ and its interaction partners. Since the two networks (A^+^ and A^-^) represent different prognosis statuses, genes with large PRV values are considered potential prognostic biomarkers. These are then included as the gene feature subsets for building the following classifiers.

In summary, we chose 20 prognostic biomarkers for breast cancer, and 10 clinical features were adopted in our experiments, including age, menopausal state, tumor size, radiotherapy, chemotherapy, hormone therapy, neoplasm histologic grade, cellularity, surgery-breast conserving, and surgery-mastectomy (Tables S1 & S2). On the other hand, we selected 15 prognostic biomarkers for non-small cell lung cancer and included age, gender, and tumor stages in our analyses (Tables S1 & S3). We have already detailed the biomarker selection processes in our previous papers, and their biological insights were also discussed^3,4^.

**C. Robust semi-supervised cancer prognosis classifier with Bayesian variational autoencoder (*SCAN*)**

Under our experimental settings, all patients come with complete gene expression profiles of selected prognostic biomarkers. *SCAN* can thus make at least a coarse prediction based on only the microarray profiles ($x$). Considering whether there are missing values in clinical data ($c$) or labels ($y$), we can divide all patients into four different types. Type I patients have complete clinical information and their corresponding labels ($x, c, y$). Type II patients have complete clinical information but are without their corresponding labels, resulting from censorship or insufficient event information (with or without event and event time) ($x_{u}, c_{u}$). They consist of the majority of the cohort. The third type of patients (Type III) do not have complete clinical information or labels, including censored patients and those with only microarray data ($x_{x}$). Lastly, Type IV patients have missing values in their clinical information, but the corresponding labels can be defined according to their event occurrence and event time. Note that no Type IV patients were identified in both the considered breast or non-small cell lung cancer cohorts in this study. However, *SCAN* can be easily generalized to include Type IV patients.


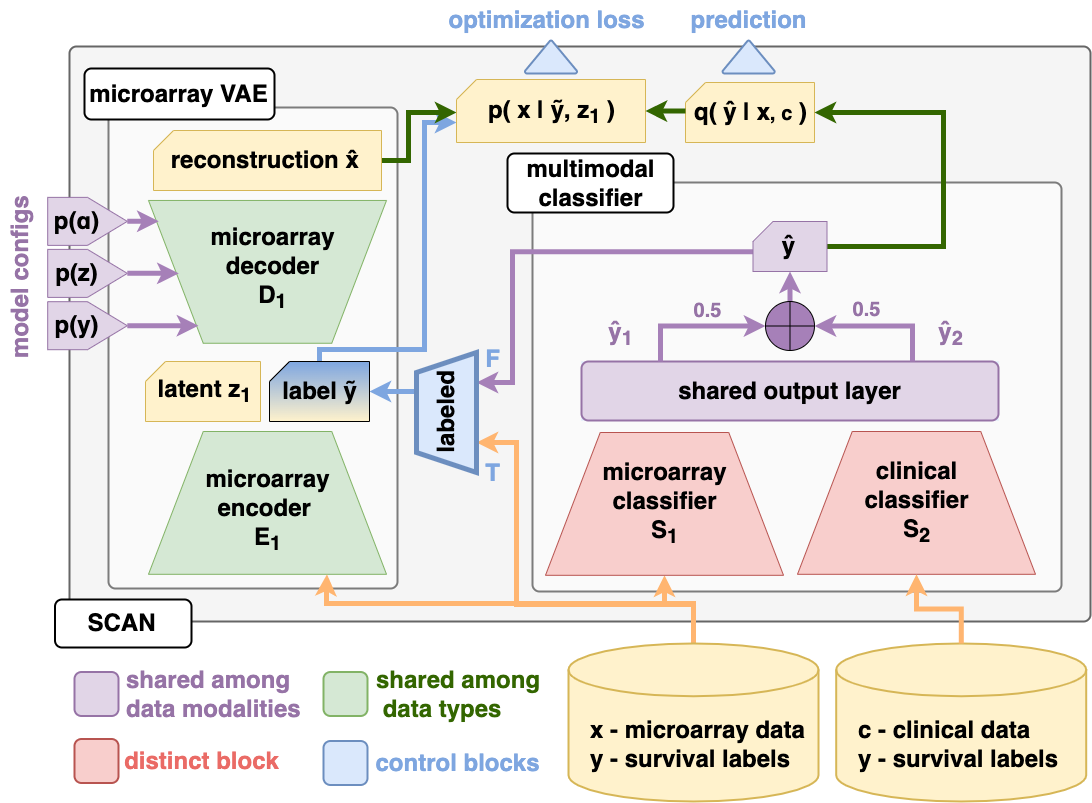


**Figure S1. The architecture of *SCAN*.**

The *SCAN* framework generalizes the M2 architecture^7^ by making the final prediction with majority votes from subnetwork classifiers and designing objective loss functions tailored for all patients. Although we did not list the loss function for Type IV patients here, one can follow the general idea mentioned below to include it in the overall loss function. The architecture of *SCAN* is illustrated in Fig. S1. In addition to a microarray encoder paired with a microarray decoder, *SCAN* includes a microarray and clinical classifiers followed by a shared output layer to generate the final prediction. The multimodal classifier here reads in both microarray and clinical data. The outputs from $S_{1}$ and $S_{2}$ are then weighted by the shared output layer. The weighted outputs $\hat{y}_{1}$ and $\hat{y}_{2}$ are combined with equal importance to generate the final prediction $\hat{y}$. We can formulate the shared output layer as the following equation:

$\hat{y}_{i}= \sigma\left( {\boldsymbol{1}_{\boldsymbol{x}}\boldsymbol{\odot w}}_{\boldsymbol{x}}^{T}\boldsymbol{o}_{\boldsymbol{x}}+ \boldsymbol{1}_{\boldsymbol{c}}\boldsymbol{\odot}\boldsymbol{w}_{\boldsymbol{c}}^{T}\boldsymbol{o}_{\boldsymbol{c}} \right), i=1, 2,$ (5)

where $\boldsymbol{o}_{\boldsymbol{x}}$ and $\boldsymbol{o}_{\boldsymbol{c}}$ are the output from $S_{1}$ and $S_{2}$, respectively; $\boldsymbol{w}_{\boldsymbol{x}}$ and $\boldsymbol{w}_{\boldsymbol{c}}$ are the corresponding weights given in the shared output layer, respectively; $\boldsymbol{\odot}$ represents element-wise product; and $\sigma\left( \cdot\right)$ is the sigmoid function. The indicator functions ($\boldsymbol{1}_{\boldsymbol{x}}$ and $\boldsymbol{1}_{\boldsymbol{c}}$) make sure that the information constructing the weighted vote (prediction) is only from either microarray or clinical data. This enables patients with and without missing clinical features to jointly contribute to the overall prediction. For Type I patients, the prediction is the average of $\hat{y}_{1}$ and $\hat{y}_{2}$ from (5). The prediction is directly the $\hat{y}_{i}$ from the available subnetwork classifier for the other types of patients. For instance, predictions for Type III patients are generated from the microarray subnetwork classifier only since no clinical information is available.

For the microarray VAE part, a dedicated loss function is designed for each type of patient. For Type I patients, the evidence lower bound (ELBO) of the data generating probability can be formulated as follows^7^:

$p\left( x, y \right) \geq E_{q\left( z | x,y \right)}\left[ \log p\left( x | z,y \right)+ \log p\left( z \right)+ \log p\left( y \right)- \log q\left( z | x,y \right) \right],$ (6)

which is identical to that proposed in the original paper^7^. In (6), $q\left( z | x,y \right)$ is the learned VAE latent distribution; $p\left( z \right)$ and $p\left( y \right)$ is the prior distribution of the VAE latent representation and patient labels, respectively. For Type II patients, the data-generating probability is then characterized by:

$p\left( x,c \right) \geq E_{q\left( z,\hat{y} | x,c \right)}\left[ \log p\left( x,c,\hat{y} | z \right)+ \log p\left( z \right)+ \log p\left( \hat{y} \right)- \log q\left( z,\hat{y} | x,c \right) \right],$ (7)

where the prediction from the multimodal classifier $\hat{y}$ is now the majority vote from $S_{1}$ and $S_{2}$ originated from (5). According to the derivations made by Kingma *et al.*, (7) can be viewed as a weighted average of (6) over the patient labels by the predicted class probabilities^7^. Based on the generated pseudo-label $\hat{y}$, we approximate the lower bound (7) by summing up the lower bounds calculated from the microarray and clinical VAEs, respectively. In our model design, we did not include a clinical VAE, so the lower bound in (7) can be further simplified by neglecting $c$. Note that the information contained in clinical data is not dropped as the pseudo-label $\hat{y}$ is generated from the majority votes from both subnetwork classifiers. As for Type III patients, we can derive a similar lower bound, but the prediction $\hat{y}$ only includes information from $S_{1}$ since only microarray data are available:

$p\left( x \right) \geq E_{q\left( z,\hat{y} | x \right)}\left[ \log p\left( x,\hat{y} | z \right)+ \log p\left( z \right)+ \log p\left( \hat{y} \right)- \log q\left( z,\hat{y} | x \right) \right].$ (8)

Now let the right-hand-side of (6), (7), and (8) be denoted as $-L(x, y)$, $-U_{xc}(x_{u}, c_{u})$, and $-U_{x}(x_{x})$, respectively, as the corresponding ELBOs of the data generating distributions. In order to update the subnetwork classifiers with Type I patient data, we followed the paper by Kingma *et al.* and added an auxiliary loss function to the subnetwork classifiers:

$BCE\left( x,c,y \right)=E_{Type I}\left[ \log p\left( y | x,c \right) \right],$ (9)

where $p\left( y | x,c \right)$ is the learned predictive distribution and BCE stands for binary cross-entropy loss. The overall loss function is then the sum of these three lower bounds and an additional cross-entropy loss for the Type I patients:

$$L= \sum_{Type I} L\left( x, y \right)+\sum_{Type II} U_{xc}\left( x_{u}, c_{u} \right)+\sum_{Type III} U_{x}\left( x_{x} \right)$$

$-\beta\sum_{Type I} BCE\left( x, c, y \right),$(10)

where $\beta$ is an adjustable coefficient. The optimal neural network weights are found by minimizing (10). Last but not least, there are two facts worth mentioning. The first one is that if we have Type IV patients in the cohort, another lower bound $U_{c}$ can be included simply by replacing $x$ with $c$ in (8). Secondly, one can weigh each lower bound differently based on domain knowledge. We think they are interesting topics to look into and are listed as future works.

The loss function can be updated through simple back-propagation with mini-batches by the reparameterization trick. Note that the microarray VAE is jointly trained with the multimodal classifier during training. We only utilized the trained multimodal classifier during testing and independent validation for prediction.

**D. 4-fold cross-validation parameters**

4-fold cross-validation (4-CV) was performed to select model hyper-parameters of *SCAN*. The best performing set of model hyper-parameters in terms of average validation AUROC over four splits was chosen. The final model was then trained on the joint training set (with unlabeled patients) and tested on the hold-out test set. The optimizer is RMSprop^8^, as suggested in the original semi-supervised VAE paper^7^, with a learning rate of 0.01 for both cancers. The classifier regularization constant ($\beta$) and the L2-regularization constant were set to 0.1. The mini-batch sizes were set to 64 and 32 for breast and non-small cell lung cancer, respectively. Priors of all variational parameters were set to standard Gaussian distribution. The width and depth of the hidden layers of the encoder, decoder, and classifiers (microarray/clinical hidden), as well as the input transformation layer (decoder input) and the latent dimension of the AE (AE latent), were selected through a four-fold CV. Additional layers merge the (predicted) labels and latent codes from the encoder as the decoder input layers. They are followed by hidden layers of the decoder. The chosen model hyper-parameters were bold-faced, as summarized in Table S2.

The activation function of the encoder and decoder networks is the exponential linear unit (ELU)^9^, and the other neural networks adopted softplus^10^ as their activation function. The output predicted probability was generated from a sigmoid activation layer. As for the other models, such as *Bimodal*, support vector machine (SVM), and random forest (RF), we followed the same sets of hyper-parameters in our previous research^3,4^.

**Table S2. Model hyper-parameter selection.** Since different depths of neural networks were trained, we used ‘N’ to represent shallow networks that do not have those layers.

|  | breast | NSCLC |
| --- | --- | --- |
| encoder hidden 1 | 15, **10** | **12** |
| encoder hidden 2 | **10**, 5, N | 10, 8, **N** |
| encoder hidden 3 | **5**, N | 8, **N** |
| decoder hidden 1 | **5**, 10 | **8** |
| decoder hidden 2 | 10, 15, **N** | 10, 12, **N** |
| decoder hidden 3 | 15, **N** | 12, **N** |
| microarray hidden 1 | **10** | **12** |
| microarray hidden 2 | **5**, N | 8, **N** |
| microarray hidden 3 | **N** | 4, **N** |
| clinical hidden 1 | **10** | 3, **N** |
| clinical hidden 2 | 5, **N** | 3, 2, **N** |
| decoder input | 10, **5** | **10**, 5 |
| AE latent | **10**, 5 | 10, **5** |
| epochs | 50, 100, 150, **200**, 300 | 50, **100**, 150, 200, 300 |

**E. Additional materials and methods**

**E.1 Variational Inference and regularization**

In probabilistic modeling literature, neural networks are commonly used to depict the complex interactions between observed and latent variables (*p-model*). A complementary set of neural networks (*q-model*) are also included to approximate the intractable data posterior. For instance, the variational autoencoder^11^ models the relation between the latent codes and the input features with a decoder (*p-model*). The distribution of the latent codes is captured by the encoder (*q-model*). Probabilistic models are updated by maximizing the joint distribution of the observed and latent variables. However, such optimization is often intractable. Thus, certain assumptions on how the *q-model* factorizes, such as the mean-filed^12^ approach, are imposed such that the joint distribution is easy to compute. With the factorized *q-model* distribution, the evidence lower bound (ELBO) serves as a surrogate objective function for maximization, and the neural networks are updated through simple back-propagation^11^. Neural networks are susceptible to overfitting on small datasets, and specific regularization approaches such as L1/2-regularization are available to constrain the neural network parameter growth and reduce potential overfitting^13,14^. All neural networks were implemented in *SCAN* with L2-norm constraints on their parameters. Furthermore, we implemented the full variational Bayes (Full VB) version of VAE as mentioned in D.-P. Kingma & M. Welling’s paper^11^, where the standard deviation of the parameters was further regularized, and the variational dropout technique was used^15^.

**E.2 Semi-supervised variational autoencoder**

VAEs are powerful deep learning modules widely utilized to extract meaningful representations from input data in an unsupervised fashion^11^. Recently, by introducing an auxiliary classifier, the so-called M2 model demonstrates the possibility of training VAEs jointly with both labeled and unlabeled data under semi-supervised learning setting^7^. Labeled patient data are hard to acquire in biological applications, and abundant unlabeled ones are relatively easier to obtain. If utilized carefully, the unlabeled data may further enhance our previous models^3,4^ by a significant margin in prediction accuracy, model robustness, and even flexibility. We made a few modifications so that *SCAN* can make predictions based on either microarray or clinical data, or even both in an ensemble fashion. The above property enables researchers to use censored patient data and those with missing clinical records. Both types of data are often encountered in practical application scenarios.

**E.3 Performance evaluation metrics**

We included five representative performance evaluation metrics in all comparisons to have a comprehensive model performance comparison, including AUROC, macro F1, CI, AUPRC, and ACC. Our main performance evaluation metric, AUROC, is the area under the ROC curve, and larger AUROC scores indicate stronger prediction power^16^. However, the common pitfall of AUROC is that it cannot reflect the class imbalance issue in the data and fails to discriminate against bad predictions^17^. Considering this issue, we also included the complementary metric as the F1-score, the geometric mean of the precision and recall scores^18^. To examine whether the classifier makes predictions biased toward certain classes, the macro F1-score computes the F1-score of each class and uses an unweighted average to retrieve the final score. Macro F1 is widely used in recent deep learning research as a robustness measurement^19,20^. In addition to the Kaplan-Meier (KM) analysis, CI is also a commonly used metric that measures the proportion of predicted risk scores in accordance with the actual survival time^21^. AUPRC, also known as the average precision (AP)^22^, averages the precision scores at every threshold (or every possible recall score) and is the area under the precision-recall curve. Although this metric can reflect the label imbalance issue, we are more focused on one of the operating points with a specific threshold, and thus, the macro F1 score seems to be a better fit compared to AUPRC. The last metric included in the comparison is ACC. It measures the proportion of correctly classified samples. However, it is also known to be highly sensitive to class imbalance and thresholding^23^. In our experiments, we mainly focused on AUROC and CI.

**E.4 Bootstrap confidence intervals**

The empirical bootstrap is a statistical technique popularized by Bradley Efron in 1979 to estimate the variations of statistics measured on the same data^24^. It is, therefore, an appropriate approach for estimating the confidence intervals of the model performance metrics. Given a set of data drawn from an unknown data-generating distribution $F$, the bootstrap setup starts with calculating an estimate $u$ from the data. An empirical bootstrap sample is a resample (sampling with replacement) of the same size from the given data, regarded as the samples drawn from the empirical distribution $F^{*}$ (also called the resampling distribution). From the empirical bootstrap sample, we can compute the estimate as $u^{*}$. The empirical bootstrap setup repeats the resampling process for $B$ times, and the variations of $u^{*}$ can be estimated. As long as $B$ is large enough, the variation of $u$ is well-approximated by the variation of $u^{*}$. We can obtain the confidence intervals in this manner. In this work, we created 1000 bootstrap test sets and obtained the 95% confidence intervals of all model performance metrics such as AUROC, AUPRC, macro F1, CI, and ACC.

**F. Ablation study**

To examine the contribution of each block, we performed ablation studies by removing the corresponding loss functions. We summarized the results in Tables S3 and S4. The rows represent the prediction performance of each subnetwork classifier ($y_{x}$, $y_{c}$) and the majority vote ($y_{xc}$). The columns represent different cases of ablation studies. The first column (ALL) is the performance of the original *SCAN* model. In the second column (-III), the contribution of Type III patients was removed such that $U_{x}\left( x_{x} \right)$ does not contribute to the model update. Then, the third column (-III -II) further removes contribution from $U_{xc}\left( x_{u}, c_{u} \right)$ (Type II patients). Lastly, the fourth column (-III -II - I) further removes the microarray VAE loss ($L\left( x, y \right)$) of Type I patients, showing the performance of solely the subnetwork classifiers. Throughout the ablation study, the model hyper-parameters were held unchanged, and we retrained the model with the modified loss functions as detailed above. As the hyper-parameters were optimized with all Type I-III patients, we expect the altered model to encounter performance fluctuation as we remove more loss functions. Better models could be obtained by performing 4-CV with the altered loss functions.

We observed a significant and consistent performance drop for breast cancer when removing the losses. By removing the contribution from the unlabeled data, the resulting AUROC of $y_{xc}$ dropped from 81.73% to 79.68% by approximately 2%. The AUROC decreased again by 2% when the VAE was removed (77.74%). We observed similar patterns for all five metrics. This indicates that the performance improvement mainly stems from unlabeled patient data with the microarray VAE. As a result, we could expect further performance improvement as more unlabeled patient data were included in the training process. We showed that the resulting AUROC and AUPRC scores worsened by reducing the available unlabeled data during training. As one of our future works, we plan to apply *SCAN* to even more extensive data cohorts such as TCGA.

We also performed similar analyses for NSCLC. From Table S4, we observed that the overall performance generally improved as we removed the microarray VAE. This might seem to contradict at first. However, we should note that the number of unlabeled patients is only 102 for this cohort. Implementing a complicated VAE may only help retrieve values from unlabeled data marginally. The unnecessary model complexity outweighs the potential benefits, resulting in a worse overall performance. On the other hand, the subnetwork classifiers showed that decent results could be obtained with only labeled data via the power of ensemble learning. In the last column in Table S4, $y_{xc}$ performed the best compared to $y_{x}$ and $y_{c}$. This supported our model design which generates the final prediction with a majority vote. Worse-performing subnetwork classifiers could benefit from each other and jointly produce better results. A similar trend could also be identified for breast cancer.

**Table S3. Ablation study for breast cancer prognosis prediction.**

|  | metrics | ALL | - III | - III - II | - III - II - I |
| --- | --- | --- | --- | --- | --- |
| $y_{xc}$ | AUROC (%) | **81**.**73 (7**.**98)** | 81.61 (7.96) | 79.68 (8.34) | 77.74 (8.36) |
|  | macro F1 (%) | 72.55 (8.33) | **74**.**34 (7**.**98)** | 71.79 (8.06) | 74.34 (7.93) |
|  | CI (%) | **69**.**02 (4**.**43)** | 68.84 (4.31) | 68.13 (4.56) | 67.23 (4.90) |
|  | AUPRC (%) | 77.01 (11.72) | **77**.**05 (11**.**77)** | 74.34 (12.11) | 74.84 (11.51) |
|  | ACC (%) | 72.65 (8.55) | **74**.**36 (7**.**69)** | 71.79 (7.69) | 74.36 (8.12) |
| $y_{x}$ | AUROC (%) | 79.71 (8.21) | **79**.**85 (8**.**33)** | 78.01 (8.36) | 77.16 (8.34) |
|  | macro F1 (%) | 70.05 (7.83) | 69.17 (8.13) | **70**.**84 (7**.**95)** | 69.23 (8.32) |
|  | CI (%) | 68.79 (4.69) | **68**.**82 (4**.**60)** | 67.10 (4.61) | 66.24 (4.96) |
|  | AUPRC (%) | **76**.**38 (11**.**76)** | 76.22 (11.82) | 75.01 (11.58) | 74.80 (11.38) |
|  | ACC (%) | 70.09 (7.69) | 69.23 (8.12) | **70**.**94 (7**.**96)** | 69.23 (8.55) |
| $y_{c}$ | AUROC (%) | **69**.**74 (9**.**42)** | **69**.**74 (9**.**39)** | 69.06 (9.50) | 68.39 (9.65) |
|  | macro F1 (%) | 60.36 (8.59) | 58.36 (9.40) | **64**.**92 (8**.**56)** | 64.10 (8.58) |
|  | CI (%) | 60.47 (5.50) | **60**.**70 (5**.**64)** | 60.42 (5.47) | 59.54 (5.63) |
|  | AUPRC (%) | 71.88 (11.00) | 71.99 (10.87) | **72**.**14 (10**.**86)** | 70.35 (11.21) |
|  | ACC (%) | 61.54 (8.55) | 59.83 (8.97) | **64**.**96 (8**.**55)** | 64.10 (8.55) |

**Table S4. Ablation study for NSCLC prognosis prediction.**

|  | metrics | ALL | - III | - III - II | - III – II - I |
| --- | --- | --- | --- | --- | --- |
| $y_{xc}$ | AUROC (%) | 80.46 (6.61) | 80.49 (6.63) | 80.54 (6.61) | **82**.**30 (6**.**20)** |
|  | macro F1 (%) | **72**.**70 (7**.**18)** | 72.17 (7.30) | 66.81 (7.34) | 71.52 (7.27) |
|  | CI (%) | 61.03 (4.70) | 61.05 (4.74) | **61**.**25 (4**.**67)** | 59.57 (4.83) |
|  | AUPRC (%) | 60.83 (12.85) | 60.92 (12.91) | 58.74 (13.52) | **66**.**61 (12**.**04)** |
|  | ACC (%) | 74.85 (6.73) | 74.27 (6.73) | 70.76 (6.44) | **76**.**02 (6**.**44)** |
| $y_{x}$ | AUROC (%) | 80.75 (6.63) | 80.83 (6.58) | 80.85 (6.56) | **82**.**19 (6**.**15)** |
|  | macro F1 (%) | **72**.**12 (7**.**20)** | **72**.**12 (7**.**20)** | **72**.**12 (7**.**20)** | 71.52 (7.27) |
|  | CI (%) | 59.31 (4.84) | 59.33 (4.90) | 59.34 (4.84) | **59**.**46 (4**.**94)** |
|  | AUPRC (%) | 62.67 (12.87) | 62.78 (12.93) | 62.52 (13.22) | **64**.**85 (12**.**80)** |
|  | ACC (%) | **76**.**02 (6**.**14)** | **76**.**02 (6**.**14)** | **76**.**02 (6**.**14)** | **76**.**02 (6**.**44)** |
| $y_{c}$ | AUROC (%) | 68.90 (8.56) | **69**.**21 (8**.**45)** | 69.06 (8.51) | 67.32 (9.23) |
|  | macro F1 (%) | **61**.**80 (7**.**27)** | 61.30 (7.38) | 61.30 (7.38) | 61.77 (7.66) |
|  | CI (%) | **62**.**85 (4**.**56)** | 62.78 (4.55) | 62.78 (4.55) | 61.14 (4.52) |
|  | AUPRC (%) | 43.82 (12.52) | **44**.**09 (12**.**67)** | 44.07 (12.52) | 43.92 (13.06) |
|  | ACC (%) | 64.33 (7.02) | 63.74 (7.02) | 63.74 (7.02) | **64**.**91 (7**.**32)** |

**G. VAE latent representation superposition**

Autoencoder is an unsupervised learning model helpful for extracting low-dimensional latent representation of high-dimensional data at input^11^. Every data point is mapped to a specific latent representation by minimizing the reconstruction error. The main difference between classical autoencoder and its variational counterpart lies in the latent representation modeling strategies^11^. In VAEs, the latent representations are modeled as Gaussian distribution random variables with learnable means and standard deviations. We can view each dimension of the latent representation in a VAE as independent coordinates for specific features of the input data^25^. Another research regarded a VAE as a “distributed representation” of the Gaussian mixture model (GMM)^26^. The amplitude of the latent representation controls the weights of the Gaussian distributions utilized to reconstruct the original data at the decoder output^25^. The means and the standard deviations of the Gaussian distributions are learned by the decoder^11^. With this interpretation, novel data points (from unseen class) can be automatically mapped to certain interpolation/extrapolation with the seen data points. Similar data points are mapped to latent representations close to each other.

This property is beneficial when we need to make inferences on novel samples without re-training the model. We selected two of the elements in the learned microarray VAE latent representations for demonstration, and the relationship between the predicted logits and the representation was examined. As illustrated in Fig. S2 (left), the 10^th^ latent representation was taken out for analysis. We marked the corresponding latent values of the patient with the smallest predicted survival probability as the red line. As expected, the patient lay in one of the peaks of the poor prognosis patients. Similarly, we marked the corresponding latent representation value of the patient with the highest predicted survival probability as the green line located near the peak of the good prognosis patients. Then, we selected the patient with the median predicted survival probability and marked the patient with blue in the latent space. As observed in Fig. S2 (left), the latent representation for median survival patients lay near the poor prognosis patients, where poor and good prognosis patients shared overlap, which verified that the learned latent representations in the microarray VAE did reflect the predicted death probability. Similar analyses were also performed on non-small cell lung cancer patients, and similar results were observed. The results are illustrated in Fig. S2 (right).

| 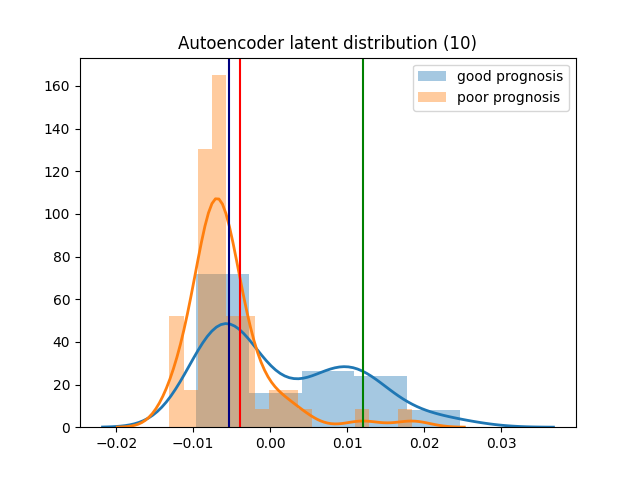 | 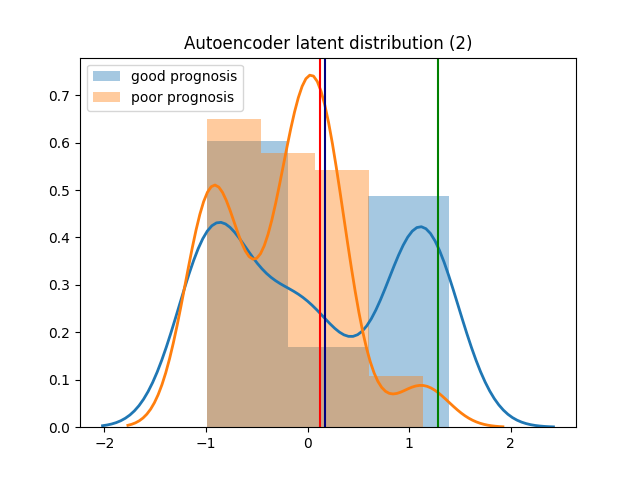 |
| --- | --- |

**Figure S2. VAE latent representation superposition (left: breast cancer; right: NSCLC).**

**H. Feature importance – connection weights algorithm**

The prognostic biomarkers, selected by our systems biology feature selector, along with the clinical features provide strong prediction power for *SCAN*. In our previous studies^3,4^, we discussed their biological meanings in-depth. In this discussion, we want to shed some light on how *SCAN* used these genomic and clinical features to yield risk predictions. In the literature, several approaches were proposed to extract the crucial features affecting model predictions. For instance, the random forest classifiers quantify feature importance via the normalized reduction in the Gini index in the out-of-bag (OOB) estimate^27^. Several other approaches were reviewed in a survey, such as generating feature importance rankings according to trained model weights, partial derivatives, stepwise selection, etc.^28^ Among them, the connection weights algorithm^29^ was reported to perform the best by matching the correct order of feature importance in the simulated data. The connection weights algorithm quantifies feature importance by accumulating the multiplication of neural network weights on every possible path from the feature to the output. As a result, the features with the largest feature importance have the most prominent effect on risk prediction. In addition to the connection weights algorithm, we also adopted the second effective approach, partial dependence plots, in the survey^28^. The results were similar and they were summarized in *Supplementary I*. The results were summarized in Figures S3 and S4.

For breast cancer (Figure S3), genomic features such as ESR1, PGR, BRTC, YWHAQ, and PLK1 were considered crucial for risk prediction as they achieved the top 25% absolute feature importance scores. ESR1 is a well-known breast cancer biomarker included in one of our split criteria when building gene interaction networks. It was shown in a recently published research that the prevalence of ESR1 mutations in local recurrence carries a worse prognosis^30^. PGR was also found to be a significant gene biomarker that can serve as a potential prognostic indicator^31^. It was reported that BTRC is related to the disease-free and overall survival of breast cancer patients^32^. YWHAQ was identified to be both closely related to breast and non-small cell lung cancer prognosis^33,34^. Last but not least, PLK1 overexpression has been found in many cancers to be associated with poor cancer prognosis^35^. As for the clinical features, we found that whether the patient has gone through chemotherapy and hormone therapy and the tumor size significantly affect the prognosis prediction. It was reported that different breast cancer molecular subtypes show distinct sensitivities to preoperative chemotherapy^36^. According to research, high *resistin* expression in breast cancer tissues correlates significantly with tumor size, and hormone therapy decreases the recurrence rate in patients with high *resistin* expressions^37^, which is aligned with our model’s observation.

| 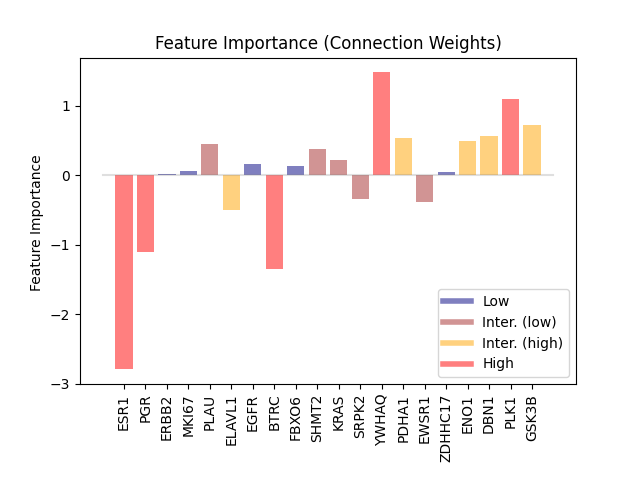 | 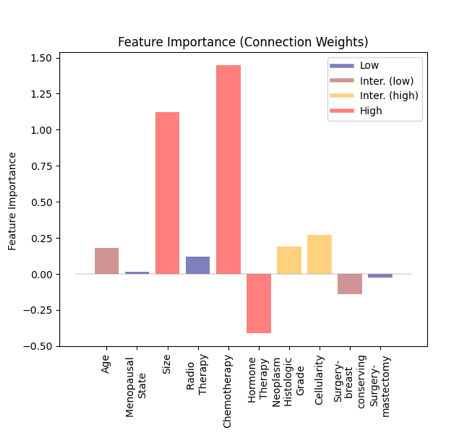 |
| --- | --- |

**Figure S3. Microarray (left) and clinical (right) feature importance (connection weights algorithm; breast cancer).**

We also applied the connection weights algorithms to identify the essential features for NSCLC prognosis prediction. As shown in Figure S4, we found that CADM1, SLC2A1, ELAVL1, and NRF1 were considered crucial. They were also recognized as essential prognostic factors. A five-biomarker panel, including both CADM1 and SLC2A1 was analyzed by immunohistochemistry tissue microarrays where the biomarkers were selected based on their prognostic association and relation to diverse biological processes^38^. Moreover, both ELAVL1 and NRF1 achieved the top-three PRV scores in all PRV lists for all pairs of GINs for all well-known biomarkers. Interested readers are suggested to visit our previously published paper for a more detailed discussion^3^. For the clinical features, we observed that the cancer stage was found to be essential for prediction. It was suggested that tumor stage, performance status, and age are the best predictors of overall survival, which are utilized to guide therapy in clinical practice^39^. Research results showed that both stage and gender were considered significant independent variables among the other prognostic factors^39^. Although only the stage achieved significantly larger importance among the three clinical features used, we can also observe that age achieved a more significant score compared to gender, which is aligned with the results mentioned above. The feature importance of *SCAN* reported via the connection weights algorithm is consistent with much existing medical research. This further validates the interpretability of *SCAN*.

| 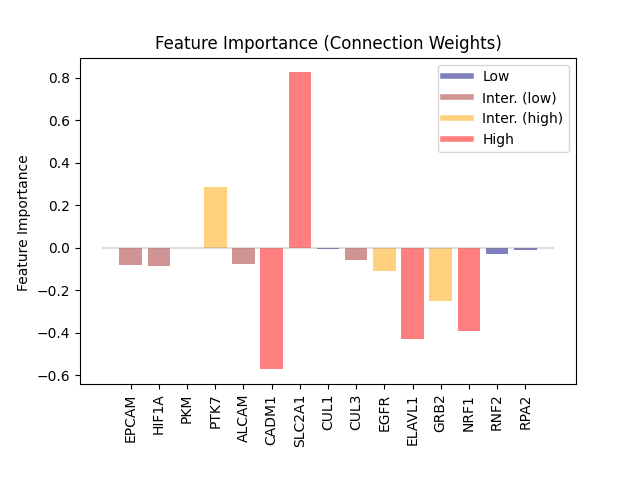 | 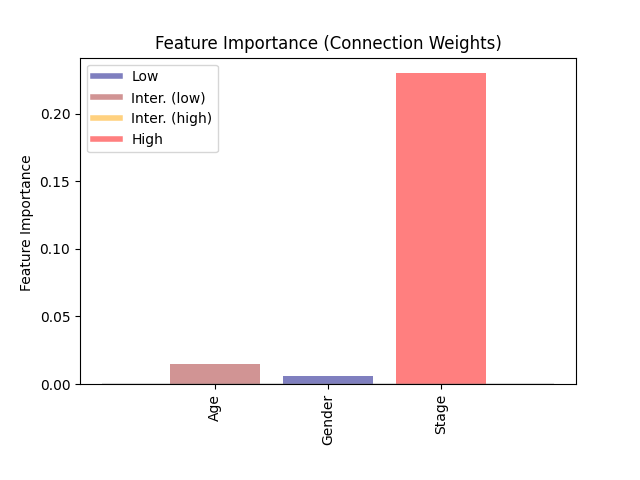 |
| --- | --- |

**Figure S4. Microarray (left) and clinical (right) feature importance (connection weights algorithm; NSCLC).**

**I. Feature importance - partial dependence plots (PDPs)**

We further follow the concepts of PDPs and measure the difference in the obtained AUROC scores to verify the essential features suggested by the connected weights algorithm^40^. The PDPs for each feature were drawn by changing the target feature values for all patients from the minimum to the maximum while keeping other feature values unchanged. Features with large absolute AUROC score differences are considered important, as their values may greatly impact the overall prediction. In brief, we obtained similar sets of essential features from the connection weights algorithm.

In Figure S5, we plotted the PDPs based on the subnetwork classifiers for breast cancer patients and summarized the maximum changes in AUROC as feature importance. Similar trends can be observed when comparing the feature importance obtained from the connection weights algorithm and the differences between AUCs from PDPs. As for NSCLC, as illustrated in Fig. S6, we found that SLC2A1 was still recognized as one of the important gene biomarkers and observed that stage remained one of the top crucial clinical features.

| 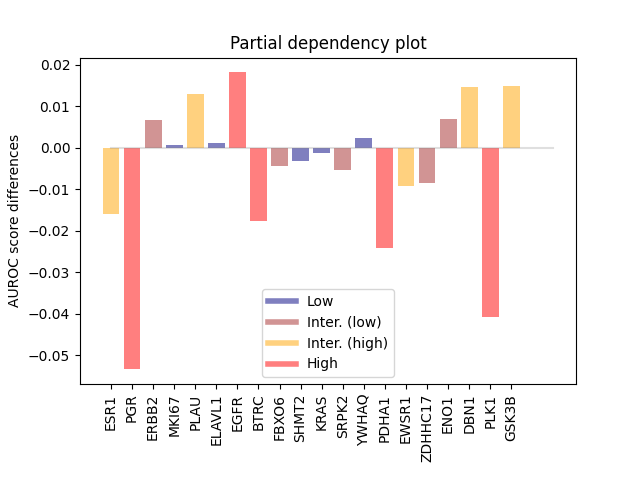 | 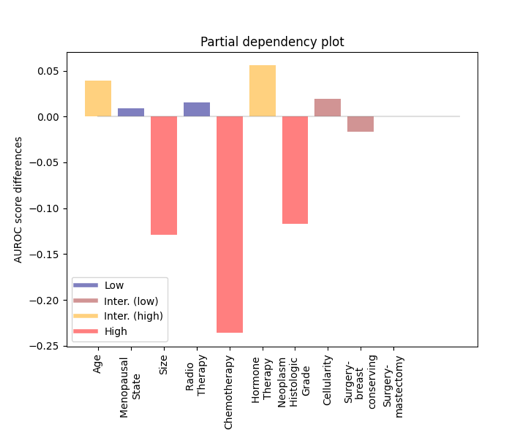 |
| --- | --- |

**Figure S5. Microarray (left) and clinical (right) feature importance (PDP; breast cancer).**

| 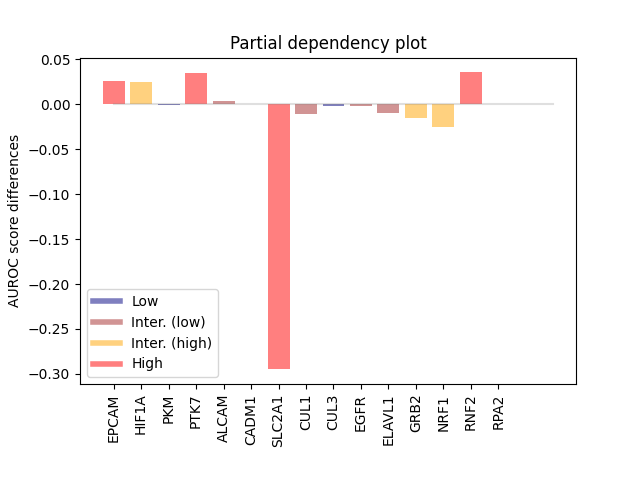 | 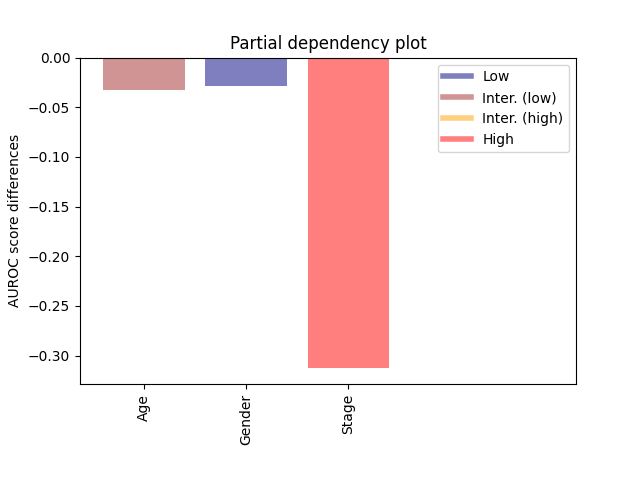 |
| --- | --- |

**Figure S6. Microarray (left) and clinical (right) feature importance (PDP; NSCLC).**

**J. External validations on TCGA cohort**

In order to further validate the robustness of *SCAN*, we collected breast (TCGA-BRCA) and non-small cell lung cancer (TCGA-LUAD) patient data from TCGA. We used the model trained with the joint training sets and directly tested on the two TCGA datasets. The results are summarized in Table S5. For breast cancer, since we are not able to find breast cancer patients from TCGA-BRCA with the full clinical features as used in the METABRIC dataset, we can only test our model on the microarray subnetwork classifier ($y_{x}$). As we can observe from the first row of results in Table S5, due to the drastic different data distribution, normalization methods, and label ratio (poor-to-good prognosis) changes, the microarray subnetwork classifier performed similarly to random guess with AUROC and AUPRC approximately equal to 50% (random guess) and 10% (label ratio). To improve this, we may need to apply minority class-aware loss functions such as the balanced class weight or focal loss^41^.

On the other hand, for NSCLC (TCGA-LUAD), we observed similar results for the microarray subnetwork classifier. However, we observe a much better performance for the clinical subnetwork classifier. Moreover, for the final prediction generated from the majority vote from the two subnetwork classifiers, $y_{xc}$, was boosted by the prediction from the clinical subnetwork classifier, $y_{c}$, which verified the model design. For a closer look at the results for TCGA-LUAD, we identified that the clinical features contribute much more information to cancer prognosis prediction. Based on our preliminary results (unpublished), for both TCGA-BRCA and TCGA-LUAD, we found that the clinical features in both cancers provide much more information for cancer prognosis prediction. In particular, we implemented a unified bimodal neural network classifier with multitask learning to utilize patient data from multiple cancers jointly. We also found that the microarray subnetwork classifier performed much worse than the clinical one aligned with the results we observed in Table S5.

**Table S5. External validation results on TCGA.**

| Classifiers | AUROC (%) | Macro F1 (%) | CI (%) | AUPRC (%) | ACC (%) |
| --- | --- | --- | --- | --- | --- |
| $y_{x}$ (BRCA) | 55.49 (3.86) | 29.62 (2.65) | 53.45 (3.09) | 10.31 (2.06) | 31.38 (2.75) |
| $y_{x}$ (LUAD) | 50.00 (0.00) | 39.75 (1.51) | 50.00 (0.00) | 34.03 (4.18) | 65.97 (4.18) |
| $y_{c}$ (LUAD) | 67.02 (5.18) | 25.39 (2.34) | 64.15 (4.44) | 48.02 (7.58) | 34.03 (4.18) |
| $y_{xc}$ (LUAD) | 67.02 (5.18) | 26.45 (2.60) | 64.15 (4.44) | 48.02 (7.58) | 34.66 (4.18) |

**K. *SCAN* improves with more unlabeled data**

To test the model performance of *SCAN* with more (> 100%) unlabeled data, we duplicated the currently available unlabeled patient data by 1.5, 2, 3, 4, and 8 times the size of the original one. The results are summarized in Tables S6 and S7. Initially, we observed an approximate 1% improvement in all metrics if more unlabeled data were included. However, the performance started to drop as we kept duplicating the data. It even became worse than the original model when we made the unlabeled data 8 times larger than the original one. We observed similar trends like this in both breast and non-small cell lung cancers.

**Table S6. The duplicated unlabeled patient data for NSCLC prognosis prediction.**

|  | AUROC (%) | Macro F1 (%) | CI (%) | AUPRC (%) | ACC (%) |
| --- | --- | --- | --- | --- | --- |
| 1.5X | 80.88 (6.66) | 80.88 (6.66) | 70.45 (7.40) | 58.45 (4.98) | 62.55 (13.47) |
| 2X | 81.06 (6.69) | 81.06 (6.69) | 70.52 (7.19) | 59.11 (4.86) | 62.41 (13.43) |
| 3X | 80.96 (6.67) | 80.96 (6.67) | 71.05 (7.09) | 58.94 (4.89) | 64.17 (12.52) |
| 4X | 80.35 (6.66) | 80.35 (6.66) | 70.16 (7.27) | 60.59 (4.76) | 61.13 (12.75) |
| 8X | 79.99 (6.67) | 79.99 (6.67) | 67.70 (7.30) | 59.49 (4.75) | 61.39 (12.62) |

**Table S7. The duplicated unlabeled patient data for breast cancer prognosis prediction.**

|  | AUROC (%) | Macro F1 (%) | CI (%) | AUPRC (%) | ACC (%) |
| --- | --- | --- | --- | --- | --- |
| 1.5X | 81.23 (8.02) | 73.50 (8.01) | 68.69 (4.38) | 76.80 (11.70) | 73.50 (8.12) |
| 2X | 81.55 (8.02) | 71.79 (8.09) | 69.02 (4.32) | 77.01 (11.92) | 71.79 (8.12) |
| 3X | 81.14 (8.13) | 73.38 (7.90) | 68.72 (4.33) | 77.79 (11.49) | 73.50 (7.69) |
| 4X | 80.35 (8.11) | 71.76 (7.77) | 68.26 (4.46) | 77.82 (11.32) | 71.79 (7.69) |
| 8X | 71.38 (9.45) | 63.78 (8.84) | 63.03 (4.86) | 74.18 (10.32) | 64.10 (8.55) |

**L. Future works**

We list two potential future works of *SCAN* here.

**L.1. Generalization of *SCAN* to multiple heterogeneous data sources**

Numerous heterogeneous data sources can provide more helpful information for deep learning algorithms, which may help predict cancer prognosis for *SCAN*. However, if such a large set of features were inputted into a unified neural network classifier, the model complexity would increase significantly with the number of data sources. One strategy is to design distinct subnetworks to extract domain-specific features for the consequent prediction in the merged subnetwork. For instance, if we want to include additional computed tomography scan images for NSCLC five-year OS prediction, other convolutional layers need to be added to the overall model. The model complexity increases approximately linearly with the number of data sources included. However, this may result in severe overfitting in the prediction model even after a preliminary feature selection process because the number of patients remains unchanged while the number of network parameters grows linearly with the number of data modalities. Furthermore, the merged layer needs to be adjusted and retrained with the multimodal architecture every time a new data modality is included, leading to high complexity and limitation to model scalability.

*SCAN* can include new data sources without adjusting and retraining the already trained subnetworks by adopting a shared layer before generating predictions from subnetwork classifiers. The final prediction is still the majority vote from all data subnetwork classifiers. Only newly included subnetwork classifiers (possible with a VAE) must be trained. Thus, we could potentially have an arbitrary number of data sources. This significantly improves the scalability of our proposed *SCAN* architecture. *SCAN* can actually be applied to many other medical prediction tasks. Since the training time of *SCAN* is not long (usually ends in a few hundred epochs with relatively shallow networks), one can update the overall model after collecting sufficient data via knowledge distillation or transfer learning techniques. For future work, we plan to train *SCAN* on data cohorts with much more patients with heterogeneous clinical features, such as the TCGA cohort.

**L.2. Federated learning**

Federated learning^42^ was proposed as a novel distributed learning scheme where data privacy can be better preserved. Based on different data/model sharing strategies, certain variations such as FDML^43^, CoCoA^44^, and DNNC^45^ were proposed. The amount of data is rapidly increasing. Under the federated learning framework^42^, it has become impossible for a single node, such as a clinic, hospital, or academic project, to store the whole dataset locally for model training, not to mention the patient privacy issues^46,47^. Data lies in the users’ devices in distributed learning scenarios. They update their local model with the parameter server, where the users’ models are aggregated and then sent back to the users. This way, a shared model can be jointly optimized, and devices can hold their data without sharing it. Recently, federated learning has gained attraction for healthcare applications^48^. Recent research has shown that models trained with FL can perform comparably to models trained with centrally-hosted datasets^46^. FL has emerging application scenarios for healthcare applications, and it is still an active research topic in this field^47,49^.

FL can have a direct impact on healthcare applications. For instance, the HealthChain project develops an FL framework across four hospitals in France^50^. The project helps predict the treatment response of breast cancer patients. Based on the ensemble prediction architecture of *SCAN*, we can deploy the FL framework directly on our model. Under the federated learning setting, a global model will be hosted in a hospital, and several models will be hosted by collaborating institutes such as clinics. Hospitals usually have more data types. A *SCAN* model can be trained with all the available data types and the corresponding subnetwork classifiers. On the other hand, clinics may have fewer data types and sizes. To contribute to the global model, (simplified) *SCAN* models with only the available subnetwork classifiers can be trained locally. Then, the trained subnetworks can be aggregated to the global model (parameter server) under a federated learning framework. Collaborating clinics can help update the subnetwork classifiers in the hospital even with less data in this manner. The final prediction is the majority vote from all subnetwork classifiers in the global model.

In brief, the hospital and the collaborating clinics can serve as clients under a federated learning framework. They all host their own versions of *SCAN* models (simplified or full) and contribute to the powerful global model by updating different subnetwork classifiers. The resulting global model is then distributed to all clients.

**References**

1. Curtis, C. *et al.* The genomic and transcriptomic architecture of 2,000 breast tumours reveals novel

subgroups. *Nature* **486**, 346–352 (2012).

2. Pereira, B. *et al.* The somatic mutation profiles of 2,433 breast cancers refine their genomic and

transcriptomic landscapes. *Nat Commun* **7**, 11479 (2016).

3. Lai, Y.-H. *et al.* Overall survival prediction of non-small cell lung cancer by integrating microarray

and clinical data with deep learning. *Sci Rep* **10**, 4679 (2020).

4. Cheng, L.-H., Hsu, T.-C. & Lin, C. Integrating ensemble systems biology feature selection and

bimodal deep neural network for breast cancer prognosis prediction. *Sci Rep* **11**, 14914 (2021).

5. Sahoo, D., Dill, D. L., Tibshirani, R. & Plevritis, S. K. Extracting binary signals from microarray time-

course data. *Nucleic Acids Research* **35**, 3705–3712 (2007).

6. Stark, C. *et al.* BioGRID: a general repository for interaction datasets. *Nucleic Acids Research* **34**,

D535–D539 (2006).

7. Kingma, D. P., Rezende, D. J., Mohamed, S. & Welling, M. Semi-Supervised Learning with Deep

Generative Models. *arXiv:1406.5298 [cs, stat]* (2014).

8. Ruder, S. An overview of gradient descent optimization algorithms. *arXiv:1609.04747 [cs]* (2017).

9. Clevert, D.-A., Unterthiner, T. & Hochreiter, S. Fast and Accurate Deep Network Learning by

Exponential Linear Units (ELUs). *arXiv:1511.07289 [cs]* (2016).

10. Dugas, C., Bengio, Y., Bélisle, F., Nadeau, C. & Garcia, R. Incorporating second-order functional

knowledge for better option pricing. in *Proceedings of the 13th International Conference on Neural*

*Information Processing Systems* 451–457 (MIT Press, 2000).

11. Kingma, D. P. & Welling, M. Auto-Encoding Variational Bayes. *arXiv:1312.6114 [cs, stat]* (2014).

12. Saul, L. K., Jaakkola, T. & Jordan, M. I. Mean Field Theory for Sigmoid Belief Networks.

*arXiv:cs/9603102* (1996).

13. Hastie, T. Ridge Regularizaton: an Essential Concept in Data Science. *arXiv:2006.00371 [cs, stat]*

(2020).

14. Tibshirani, R. Regression Shrinkage and Selection via the Lasso. *Journal of the Royal Statistical*

*Society. Series B (Methodological)* **58**, 267–288 (1996).

15. Kingma, D. P., Salimans, T. & Welling, M. Variational Dropout and the Local Reparameterization

Trick. *arXiv:1506.02557 [cs, stat]* (2015).

16. Fawcett, T. An introduction to ROC analysis. *Pattern Recognition Letters* **27**, 861–874 (2006).

17. Saito, T. & Rehmsmeier, M. The Precision-Recall Plot Is More Informative than the ROC Plot When

Evaluating Binary Classifiers on Imbalanced Datasets. *PLoS One* **10**, e0118432 (2015).

18. Powers, D. M. W. Evaluation: from precision, recall and F-measure to ROC, informedness,

markedness and correlation. *arXiv:2010.16061 [cs, stat]* (2020).

19. Gui, T. *et al.* TextFlint: Unified Multilingual Robustness Evaluation Toolkit for Natural Language

Processing. *arXiv:2103.11441 [cs]* (2021).

20. Huang, C. *et al.* Sample imbalance disease classification model based on association rule feature

selection. *Pattern Recognition Letters* **133**, 280–286 (2020).

21. Harrell, F. E., Lee, K. L. & Mark, D. B. Multivariable prognostic models: issues in developing models,

evaluating assumptions and adequacy, and measuring and reducing errors. *Stat Med* **15**, 361–387

(1996).

22. Zhu, M. Recall, precision and average precision. (2004).

23. Longadge, R. & Dongre, S. Class Imbalance Problem in Data Mining Review. *arXiv:1305.1707 [cs]*

(2013).

24. Efron, B. Bootstrap Methods: Another Look at the Jackknife. *The Annals of Statistics* **7**, 1–26 (1979).

25. Doersch, C. Tutorial on Variational Autoencoders. *arXiv:1606.05908 [cs, stat]* (2021).

26. Bishop, C. M. Pattern recognition. *Machine learning* **128**, (2006).

27. Breiman, L. Random Forests. *Machine Learning* **45**, 5–32 (2001).

28. Olden, J. D., Joy, M. K. & Death, R. G. An accurate comparison of methods for quantifying variable

importance in artificial neural networks using simulated data. *Ecological Modelling* **178**, 389–397

(2004).

29. Olden, J. & Jackson, D. Illuminating the ‘black box’: A randomization approach for understanding

variable contributions in artificial neural networks. *Ecological Modelling* **154**, 135–150 (2002).

30. Zundelevich, A. *et al.* ESR1 mutations are frequent in newly diagnosed metastatic and loco-regional

recurrence of endocrine-treated breast cancer and carry worse prognosis. *Breast Cancer Research* **22**,

16 (2020).

31. Kurozumi, S. *et al.* Power of PgR expression as a prognostic factor for ER-positive/HER2-negative

breast cancer patients at intermediate risk classified by the Ki67 labeling index. *BMC Cancer* **17**, 354

(2017).

32. Zhang, B. *et al.* TSPAN15 interacts with BTRC to promote oesophageal squamous cell carcinoma

metastasis via activating NF-κB signaling. *Nat Commun* **9**, 1423 (2018).

33. Hou, H. *et al.* Peripheral blood transcriptome identifies high-risk benign and malignant breast lesions.

*PLOS ONE* **15**, e0233713 (2020).

34. Chang, Y.-C. *et al.* Differential expression patterns of housekeeping genes increase diagnostic and

prognostic value in lung cancer. *PeerJ* **6**, e4719 (2018).

35. Liu, Z., Sun, Q. & Wang, X. PLK1, A Potential Target for Cancer Therapy. *Translational Oncology*

**10**, 22–32 (2017).

36. Rouzier, R. *et al.* Breast Cancer Molecular Subtypes Respond Differently to Preoperative

Chemotherapy. *Clin Cancer Res* **11**, 5678–5685 (2005).

37. Lee, Y.-C. *et al.* Resistin expression in breast cancer tissue as a marker of prognosis and hormone

therapy stratification. *Gynecologic Oncology* **125**, 742–750 (2012).

38. Grinberg, M. *et al.* Reaching the limits of prognostication in non-small cell lung cancer: an optimized

biomarker panel fails to outperform clinical parameters. *Modern Pathology* **30**, 964–977 (2017).

39. Sculier, J.-P., Chansky, K., Crowley, J. J., Van Meerbeeck, J. & Goldstraw, P. The Impact of

Additional Prognostic Factors on Survival and their Relationship with the Anatomical Extent of

Disease Expressed by the 6th Edition of the TNM Classification of Malignant Tumors and the

Proposals for the 7th Edition. *Journal of Thoracic Oncology* **3**, 457–466 (2008).

40. Goldstein, A., Kapelner, A., Bleich, J. & Pitkin, E. Peeking Inside the Black Box: Visualizing

Statistical Learning with Plots of Individual Conditional Expectation. *arXiv:1309.6392 [stat]* (2014).

41. Lin, T.-Y., Goyal, P., Girshick, R., He, K. & Dollar, P. Focal Loss for Dense Object Detection. in

2980–2988 (2017).

42. Konečný, J., McMahan, B. & Ramage, D. Federated Optimization:Distributed Optimization Beyond

the Datacenter. *arXiv:1511.03575 [cs, math]* (2015).

43. Hu, Y., Niu, D., Yang, J. & Zhou, S. FDML: A Collaborative Machine Learning Framework for

Distributed Features. in *Proceedings of the 25th ACM SIGKDD International Conference on*

*Knowledge Discovery & Data Mining* 2232–2240 (Association for Computing Machinery, 2019).

doi:10.1145/3292500.3330765.

44. Smith, V. *et al.* CoCoA: A general framework for communication-efficient distributed optimization.

*Journal of Machine Learning Research* **18**, 230 (2018).

45. Teerapittayanon, S., McDanel, B. & Kung, H.-T. Distributed deep neural networks over the cloud,

the edge and end devices. in *2017 IEEE 37th International Conference on Distributed Computing*

*Systems (ICDCS)* 328–339 (IEEE, 2017).

46. Li, W. *et al.* Privacy-preserving Federated Brain Tumour Segmentation. *arXiv:1910.00962 [cs]*

(2019).

47. Xu, J. *et al.* Federated Learning for Healthcare Informatics. *arXiv:1911.06270 [cs]* (2020).

48. Rieke, N. *et al.* The future of digital health with federated learning. *npj Digit. Med.* **3**, 1–7 (2020).

49. Kairouz, P. *et al.* Advances and Open Problems in Federated Learning. *arXiv:1912.04977 [cs, stat]*

(2021).

50. Galtier, M. N. & Marini, C. Substra: a framework for privacy-preserving, traceable and collaborative

Machine Learning. Preprint at https://doi.org/10.48550/arXiv.1910.11567 (2019).
